# Population Divergence along a Genetic Line of Least Resistance in the Tree Species *Eucalyptus globulus*

**DOI:** 10.3390/genes11091095

**Published:** 2020-09-18

**Authors:** João Costa e Silva, Brad M. Potts, Peter A. Harrison

**Affiliations:** 1 Centro de Estudos Florestais, Instituto Superior de Agronomia, Universidade de Lisboa, Tapada da Ajuda, 1349-017 Lisboa, Portugal; 2 School of Natural Sciences and ARC Training Centre for Forest Value, University of Tasmania, Private Bag 55, Hobart 7001, Tasmania, Australia; B.M.Potts@utas.edu.au (B.M.P.); p.a.harrison@utas.edu.au (P.A.H.)

**Keywords:** additive genetic variance-covariance matrix, evolvability, response to selection, genetic constraint, genetic line of least resistance, quantitative genetics, wood properties, *Eucalyptus globulus*

## Abstract

The evolutionary response to selection depends on the distribution of genetic variation in traits under selection within populations, as defined by the additive genetic variance-covariance matrix (**G**). The structure and evolutionary stability of **G** will thus influence the course of phenotypic evolution. However, there are few studies assessing the stability of **G** and its relationship with population divergence within foundation tree species. We compared the **G**-matrices of Mainland and Island population groups of the forest tree *Eucalyptus globulus*, and determined the extent to which population divergence aligned with within-population genetic (co)variation. Four key wood property traits exhibiting signals of divergent selection were studied—wood density, extractive content, and lignin content and composition. The comparison of **G**-matrices of the mainland and island populations indicated that the **G**-eigenstructure was relatively well preserved at an intra-specific level. Population divergence tended to occur along a major direction of genetic variation in **G**. The observed conservatism of **G**, the moderate evolutionary timescale, and close relationship between genetic architecture and population trajectories suggest that genetic constraints may have influenced the evolution and diversification of the *E. globulus* populations for the traits studied. However, alternative scenarios, including selection aligning genetic architecture and population divergence, are discussed.

## 1. Introduction

Understanding the potential of populations to respond to selection requires a multiple-trait perspective, an issue that has become increasingly important in the face of ongoing and future global environmental change [1]. For a set of quantitative traits, the additive genetic variance-covariance matrix (**G**) within populations has a crucial importance in predicting multivariate responses to selection [2], and plays a central role in facilitating or constraining the course of adaptive evolution [3,4]. Evolutionary change is determined by the availability of genetic variation in the multivariate direction of selection, which will reflect the patterns of genetic constraint represented by the **G** matrix [5,6]. One of the first indications that adaptive diversification may be constrained by the amount and structure of genetic variation within populations was provided by Schluter [7]. Working with three-spine stickleback, Schluter [7] suggested that, during the early stages of adaptive radiation, trait means of conspecific populations under natural selection may not evolve in the direction of the greatest improvement in fitness on the adaptive landscape, but instead tend to evolve closely to the direction of the leading eigenvector of **G** (**g**_max_) in the ancestral population. This will cause the orientation of the matrix of divergence among population trait means (**D**) to be biased toward **g**_max_—the direction in a multivariate trait space that has the most genetic variance—which Schluter [7] has called the “genetic line of least resistance”. However, the distribution of genetic variation in the **G** matrix will determine how a population will respond to selection, implying that, besides **g**_max_, other multivariate axes of higher than average genetic variance can also influence the direction of evolutionary change [6,8,9]. Thus, genetic constraints due to the distribution of multivariate genetic variance in **G** can affect adaptive evolution by biasing responses to selection (and hence evolutionary trends) toward directions of trait combinations with more available genetic variance. In particular, such constraints may reflect high **G**-matrix eccentricity [10,11,12], in which case genetic variation is concentrated in fewer directions of the multi-dimensional trait space, and thus genetic variance could be insufficient for a population to evolve in the direction of selection [13]. In contrast, a uniform distribution of genetic variation across the eigenvectors of **G** may be indicative that the amount and structure of genetic (co)variances will not limit adaptation, hence supporting rapid adaptive divergence among populations under directional selection [3,4]. Subsequently to Schluter’s [7] work, several empirical studies have indicated the potential for the **G**-eigenstructure to directionally constrain the course of phenotypic evolution of closely-related species or conspecific populations [6,8,9,13,14,15,16,17,18]. The role of **G** in influencing the direction and rate of phenotypic differentiation among populations under natural selection is expected to decay over evolutionary time [7]. Yet, a close association between genetic architecture and population divergence can persist over long-time scales of millions of years, whether occurring along genetic lines of least resistance (e.g., [7,18]) or along other multivariate axes of high genetic variance besides **g**_max_ (e.g., [6,16]).

Ultimately, the predictive significance of genetic constraints in causing deviations from the optimal response to selection may depend on whether **G** remains relatively stable or changes in a predictable fashion over the evolutionary time scale of interest [7,19]. Genetic (co)variances are determined by allele frequencies and the distribution of their effect sizes [20,21,22], and thus are liable to change over time and/or space owing to the effects of natural selection, genetic drift, mutation, and migration, as well as due to the interaction among these evolutionary forces [23,24,25]. Simulation-based studies exploring the impact of interactions among these forces on the dynamics of **G** have indicated that this matrix may be stable under certain conditions and unstable in others, although a general trend emerging from these studies suggests that **G**-stability (evaluated from the effects produced on the three features—size, shape, and orientation—describing the **G**-eigenstructure) over evolutionary time may reflect some compromise between multivariate patterns imposed by natural selection and mutation, coupled with large effective population size [10,26,27,28,29,30]. Thus, the long-term stability of **G** becomes eventually an empirical question and, in this sense, comparisons among closely-related species or conspecific populations have found relatively stable **G**-matrices (e.g., conserved eigenvectors, although eigenvalues may vary; [14,24,25,31,32,33]), while other empirical studies have shown evidence for divergent (unstable) **G**-matrices [34,35,36,37]. In addition, stability of **G**-matrices may depend on the traits considered (e.g., [38]), with unstable **G**-matrices being expected for traits under mainly directional selection and stable **G**-matrices being more likely for traits under correlational (and stabilizing) selection [10,23]. The genetic basis of the traits will also determine the extent to which selection may alter **G** [21]; for traits affected by many loci and alleles per locus, with a multivariate normal distribution of effects, changes in allele frequencies in response to selection are likely to be slow [22], which may facilitate genetic (co)variances to be sustained [20].

Changing genetic architecture within diversifying populations over evolutionary time may potentially alter or conserve the relationship between major axes of genetic variation and population divergence [24]. Schluter [7] assumed that populations evolve under continuous directional selection that is random with respect to **g**_max_ (hence, the bias to the direction of divergence may be interpreted as an expectation over all possible directions of selection). However, natural selection within populations is expected to directly affect **G**-matrix evolution and stability, as an alignment of **G** with the adaptive landscape may be promoted by the landscape’s curvature and the movement of the selective optimum [23,25]. Thus, as previously noted by Schluter [7], selection rather than genetic (co)variances may underlie the relationship between genetic architecture and evolutionary trajectories. Theoretical work suggests that an alignment of **G** with the adaptive landscape can be produced when traits are under stabilizing and correlational selection [10]. Additionally, when a population evolves in response to an adaptive landscape with a steadily moving optimum (inducing directional selection driven by temporal changes in the environment, for example), the direction of peak movement may affect the evolution and stability of **G** [26]. In particular, selection can cause orientation stability of **G** over evolutionary time when the adaptive landscape has a persistent configuration producing strong correlational selection (i.e., a ridge-shaped landscape), and a multivariate population mean tracks its moving adaptive peak along the landscape’s leading eigenvector (**ω**_max_) [25,26]. This direction of peak movement may be visualized as a ridge on the adaptive landscape, which has been called the “selective line of least resistance” by Arnold et al. [23]. Selection can also have an indirect impact on **G**-matrix evolution and stability via the influence on the (co)variance matrix of mutational effects (**M**), as **M** evolves to become aligned with the adaptive landscape [27,28].

Together with large effective population size, evolutionary persistent and coordinated patterns of correlational selection, pleiotropic mutation, and peak movement can contribute to eventually stabilizing the **G**-eigenstructure within diverging populations, as **G** will tend to conform its orientation to the adaptive landscape and **M** over both intermediate and long time scales [25]. Under these circumstances, populations will tend to evolve **g**_max_ axes that are oriented toward **ω**_max_ (and thus facilitating evolution along both genetic and selective lines of least resistance; [26]), and additionally both of these directions will be aligned with the leading eigenvector of **D** (**d**_max_) [23,39], hence resulting in orientation similarity between **G** and **D**. Yet, without knowing the actual direction of selection operating on traits through evolutionary time, and particularly over long timeframes, it may be difficult to verify whether an observed relationship between genetic architecture and population divergence is due to constraints imposed by genetic (co)variance patterns or to the influence of natural selection in potentially shaping both **G** and **D** [13,40].

The present study uses wood property data from populations of the Australian forest tree *Eucalyptus globulus* Labill. to address the following objectives: (1) assess the extent to which the **G**-eigenstructure has been conserved at an intra-specific level, through the comparison of **G** matrices between two distantly-related population groups (Mainland and Island); and (2) evaluate the influence of within-population genetic (co)variation on the evolutionary divergence among populations. While these evolutionary issues have been addressed in several empirical studies with plants (e.g., [15,17,37]), studies with forest tree populations are scarce and have dealt mainly with phenological traits (e.g., [41]). Trees account for about 90% of the Earth’s biomass and, while independently evolved in many plant families, share functional features such as large size, long life span, and a self-supporting perennial woody trunk [42]. We here considered four traits commonly used to characterize their trunk wood—basic density, extractives content, lignin content, and lignin composition—which likely exhibit varying degrees and types of integration (*sensu* [43]). Basic density is a complex trait, which in angiosperms is influenced by the properties of the xylem vessels and the matrix of fibers and other cells [44]. It is often negatively associated with drought-induced mortality/damage or home-site drought conditions [45,46,47]. This association reflects an influence of underlying anatomical traits, such as vessel wall thickness and lumen area, on the vulnerability of the xylem water column to cavitation/embolism under water stress [48,49,50]. Wood extractives are a mix of non-structural compounds soluble in organic solvents or water that are mainly deposited in heartwood [51] and, as a total extract, have been positively associated with heartwood durability and decay resistance [52,53,54]. Lignin is the major structural component of the cell walls of vascular plants and is constructed from three monolignol monomers: p-hydroxyphenyl (H), guaiacyl (G), and syringyl (S) [55]. It contributes to many of the mechanical and physical characteristics of wood. Specifically, lignin confers mechanical strength, contributes to the maintenance of the water transport system within the plant (including prevention of embolism), and can act as a defensive barrier against pathogens and herbivores [55,56]. In woody angiosperms, the lignin is mainly constructed from the syringyl and guaiacyl monomers, and their ratio (S/G) varies between cell types, populations, and species [57]. Lignin rich in guaiacyl, for example, is preferentially deposited in xylem vessels, and is a more rigid and hydrophobic monomer than syringyl, although the greater elasticity of the syringyl monomer is thought to give the xylem cell walls flexibility to avoid fracture under negative pressure [56]. The lignin monomers also vary in their resistance to fungal decay, with guaiacyl being more resistant to white rot fungi than syringyl [58,59]. These fungi are one of the most common causes of wood decay in native eucalypts, entering the stem through the roots, stem damage, branch stubs, and insect galleries [59]. Lignin deposition in plant cell walls is one of the main factors that allowed the terrestrial radiation of plants [55]. Together, these four wood properties represent key functional traits in woody angiosperms impacting mechanical support, water relations, and susceptibility to pathogens and herbivores. All four traits exhibit significant genetic-based variation within and among populations of *E. globulus*, with S/G increasing, but extractives and basic density decreasing, with home-site latitude [60]. While there is evidence that divergent selection is implicated in this population differentiation [60], the extent to which the genetic architecture of the populations has influenced their evolutionary trajectories is unknown.

## 2. Materials and Methods

### 2.1. Study System and Measured Traits

*E. globulus* is native to south-eastern Australia, including the southern islands of Tasmania, where it occurs over a latitudinal range from 38.4° to 43.5° S, and altitudinal range from sea-level to 830 m [61,62]. It is one of the major eucalypts grown in industrial plantations in temperate regions of the world, including Australia [63]. During its domestication, large common-garden, base-population trials were established using open-pollinated (OP) seed collected from wild mother trees sampled in natural populations across the geographic range of *E. globulus* [64]. These field trials have shown extensive population differentiation in numerous quantitative traits, which Dutkowski and Potts [61] summarized by classifying the species into 13 geographic races and 20 subraces (the subraces are, hereafter, referred to as “populations”). The mainland and island populations are separated along a latitudinal gradient and by the major disjunction of Bass Strait, which has formed on multiple occasions following rising sea-levels during the warming phases of the Pleistocene glacial cycles [65]. The mainland populations (hereafter referred to as the “Mainland” group of populations) are broadly grouped on nuclear microsatellites, as are the eastern Tasmanian and adjacent Furneaux Island populations (hereafter referred to as the “Island” group of populations) [66,67].

We used wood property data collected from one of the largest base-population field trials of *E. globulus*, as detailed in Stackpole et al. [60,68]. In brief, the trial was established in northern Tasmania with 570 native OP families. The experimental layout of the trial was a resolvable incomplete block design with five replicates, each divided into 24 incomplete blocks of 24 families planted in two-tree plots. At age 16 years from planting, a subset of 467 families was assessed for wood properties, with one tree per family-plot sampled in most replicates, omitting minor populations. Bark-to-bark wood cores were taken at 1.1 m from each tree to estimate basic density (BD) and to provide near-infrared predictions of the percentage of Klason lignin (KL) and methanol-derived extractives (EX) on a dry weight basis, as well as the S/G ratio. Klason lignin is the conventional way lignin content is quantified by the pulp industry, it is highly correlated with total lignin (*r* > 0.9; [54]) and widely reported in plants [56]. The current study used a subset of the field-tested populations with a representation of families that are typical of the widespread distribution of the species in either the Mainland or the Island regions. In total, 10 populations, 408 families, and 1857 trees were used; the Mainland group included 4 populations, 203 families, and 929 observations; and the Island group comprised 6 populations, 205 families, and 928 observations (Table S1; Supplementary Materials).

### 2.2. Data Analysis

The following data analyses were undertaken using ASReml [69], SAS [70], and R [71].

#### 2.2.1. Estimation and Comparison of **G** Matrices for the Mainland and Island Population Groups

Evaluating whether the **G**-eigenstructure has been conserved is the first step to explore the prospect of long-term genetic constraints on phenotypic evolution. Ideally, this evaluation would be pursued within a phylogenetic framework in empirical studies, such that contemporary populations used to compare **G** matrices are sampled on a phylogeny to assess whether there is a phylogenetically-structured pattern of variation in genetic architecture [14,24]. In any case, inferences about the stability of **G** over time based on studies comparing **G**-estimates from contemporary populations assume that these populations have a common ancestral population, as well as that their genetic architectures reflect the original **G**-matrix and changes in it through time [72]. Although the phylogeny of the studied 10 populations was estimated (Methods S1 and Figure S1; Supplementary Materials), the number of families sampled per population (Table S1) would have resulted in limited precision for some **G**-estimates, which precluded to compare **G** matrices of individual populations in a phylogenetic context. Thus, to evaluate whether the **G**-eigenstructure was conserved through the evolutionary divergence of the *E. globulus* populations, **G** matrices were compared between the Mainland and Island groups, where populations in the Island group tended to be phylogenetically distant from those in the Mainland group (Figure S1). In this sense, data for each group were combined to estimate the respective **G** as a single matrix for the populations represented within the group.

Prior to analyses, the traits were mean standardized for each group (i.e., the raw data for a trait were divided by the corresponding mean within a group) to put them on a comparable scale, and thus to avoid interpretation problems due to the effects of different trait measurement scales. All measures of the focal traits are continuous, positive real numbers for which scaling in relation to the mean is both permissible and meaningful [73]. Mean-standardized **G** matrices are also suitable for the estimation, interpretation, and comparison of measures that capture the potential for evolution ([6]; see below). Variance-component modeling was then undertaken for each group by using a multivariate linear mixed model defined as

(1) $$y\;=\;Xb\;+\;Z_{f}u_{f}\;+\;Z_{b}u_{b}\;+\;e$$

where, for the four traits denoted as 1 to 4 in subscripts, $y\;=\;({y^{\prime }}_{1},\;\ldots \;,\;{y^{\prime }}_{4}{)}^{\prime }$ is a vector of mean-scaled observations; $b\;=\;({b^{\prime }}_{1},\;\ldots \;,{b^{\prime }}_{4}{)}^{\prime }$ is a vector of fixed effects; $u_{f}\;=\;({u^{\prime }}_{f_{{}_{1}}},\;\ldots \;,\;{u^{\prime }}_{f_{{}_{4}}}{)}^{\prime }$ is a vector of random OP-family effects within populations; $u_{b}\;=\;({u^{\prime }}_{b_{{}_{1}}},\;\ldots \;,\;{u^{\prime }}_{b_{{}_{4}}}{)}^{\prime }$ is a vector of random incomplete block effects; $e\;=\;({e^{\prime }}_{1},\;\ldots \;,{e^{\prime }}_{4}{)}^{\prime }$ is a vector of random residual effects; $X\;=\;diag(X_{1},\;\ldots \;,\;X_{4})$, $Z_{f}\;=\;diag(Z_{f_{{}_{1}}},\;\ldots \;,\;Z_{f_{{}_{4}}})$ and $Z_{b}\;=\;diag(Z_{b_{{}_{1}}},\;\ldots \;,\;Z_{b_{{}_{4}}})$ are incidence matrices linking a tree phenotype to the fixed and random effects; the superscript ’ denotes the transpose operation. The vector **b** included a mean term, as well as terms for the fixed effects of populations and replicates as required for each trait. Under the model specified in Equation (1), the joint distribution of the random terms was assumed to be multivariate normal with a zero-mean vector and a block-diagonal variance matrix, specified as a direct sum of variance-covariance matrices related to the effects in $u_{f}$, $u_{b}$ and e. The variance-covariance matrix was defined for the effects in $u_{f}$ as

(2) $$Var\left[\begin{array}{l} u_{f_{{}_{1}}}\\ u_{f_{{}_{2}}}\\ u_{f_{{}_{3}}}\\ u_{f_{{}_{4}}} \end{array}\right]=\;\left[\begin{array}{l} \sigma_{f_{{}_{1}}}^{2}\;\sigma_{f_{{}_{1,2}}}\;\sigma_{f_{{}_{1,3}}}\;\sigma_{f_{{}_{1,4}}}\\ \sigma_{f_{{}_{1,2}}}\;\sigma_{f_{{}_{2}}}^{2}\;\sigma_{f_{{}_{2,3}}}\;\sigma_{f_{{}_{2,4}}}\\ \sigma_{f_{{}_{1,3}}}\;\sigma_{f_{{}_{2,3}}}\;\sigma_{f_{{}_{3}}}^{2}\;\sigma_{f_{{}_{3,4}}}\\ \sigma_{f_{{}_{1,4}}}\;\sigma_{f_{{}_{2,4}}}\;\sigma_{f_{{}_{3,4}}}\;\sigma_{f_{{}_{4}}}^{2} \end{array}\right]⊗I$$

where the diagonal and off-diagonal elements refer to trait variances and covariances, respectively; I is an identity matrix of dimension $n_{f}\times n_{f}$ ($n_{f}$ = number of OP families); ⊗ denotes the Kronecker product operation. For the effects in $u_{b}$ and e, the variance-covariance matrices were defined as in Equation (2), with the dimension of I being specified by the number of incomplete blocks and observations, respectively. Restricted maximum likelihood (REML) estimates of (co)variance parameters were obtained by using the average information REML algorithm implemented in the ASReml software [69].

*E. globulus* has a mixed mating system, resulting in the OP-seed to be a mixture of selfed and outcrossed progeny [74]. Assuming an average outcrossing rate of 70% in native forests of *E. globulus*, a coefficient of relationship between OP-sibs of 0.4 has been used to get estimates of additive genetic (co)variances based on OP-progeny [63,64], instead of 0.25 as expected for pure half-sibs [75]. Estimates of additive genetic (co)variances comprising the **G** matrix were thus obtained by multiplying the REML estimates of OP-family (co)variances by 2.5 (i.e., rather than 4 as for pure half-sibs). For a given trait, the mean-standardized additive genetic variance equals the expected proportional change per generation to directional selection of unit strength (assuming selection on the trait to be as strong as on fitness itself), and thus can be interpreted as a univariate mean-scaled evolvability (hereafter denoted as $I_{A}$; [6]). Standard errors of the additive genetic (co)variances were obtained from the inverse of the Average Information matrix (i.e., after multiplying by 2.5^2^ its diagonal elements corresponding to the OP-family effects), and the Delta method was used to calculate approximate standard errors for genetic correlations estimated between the traits. Statistical significance was assessed for variance estimates via one-tailed likelihood-ratio (LR) tests [76], and for covariance and correlation estimates via two-tailed LR tests. Two-tailed LR tests were also used to assess whether parameter estimates differed significantly between the two groups, following the approach described by Shaw [77]. Although allowing evaluation of whether **G** matrices differ in regard to the particular REML estimates of (co)variances, this approach does not enable examination of matrix differences in terms of the influence of **G**-eigenstructure on the response to selection. In this sense, we used the following measures to compare the mean-standardized **G** matrices of the two groups.

Hansen and Houle [6] developed multivariate measures that capture the potential for evolution, and showed how they can be used to interpret and compare **G** matrices. In this context, we evaluated the unconditional evolvability (e(β)), the conditional evolvability (c(β)) and the autonomy (a(β)), defined for a vector β of selection gradients and a **G** matrix. We also evaluated the evolutionary “flexibility” measure proposed by Marroig et al. [78]. Given that they are calculated as functions of a specified selection gradient β, these measures may vary in different directions of the phenotypic space. In the absence of knowledge about the actual β vector acting on the traits, we computed average measures over random selection gradients distributed within a wide range of directions, as suggested by Hansen and Houle [6]. Mean values of unconditional evolvability ($\bar{e}$), conditional evolvability ($\bar{c}$), autonomy ($\bar{a}$), and flexibility ($\bar{f}$) were calculated over 5000 random selection gradients uniformly distributed in the *q*-dimensional space (in our case, *q* = 4). For $\bar{e}$, $\bar{c}$, and $\bar{a}$, this approach led to results similar to those based on analytic approximations provided by Hansen and Houle [6]. In particular, for a mean-standardized **G** matrix, $\bar{e}$ can alternatively be calculated by the average of the eigenvalues of **G** or by the average of the trait $I_{A}$ values. Further details on the estimation and interpretation of these measures are given in Table 1, and Methods S2 (Supplementary Materials).

To evaluate the difference between the Mainland and Island **G**-matrices in the direction of the response to selection, we quantified the average similarity between the matrices in their response to a set of random selection gradients (i.e., “random skewers”; [79]). Each of the two mean-standardized **G** matrices was thus subjected to the same set of 5000 unit-length random selection vectors (generated as described in Table 1), and multivariate similarity in the directions of the response to selection was evaluated by

(3) $$\bar{\varphi }\;=\mathrm{atan}\left(\frac{1}{n}\sum_{j=1}^{n}\sin\;\varphi_{j}\;,\;\frac{1}{n}\sum_{j=1}^{n}\cos\;\varphi_{j}\right)\;\frac{180}{\pi }$$

where $\bar{\varphi }$ is the mean value of the angle (converted from radians to degrees by the factor $\frac{180}{\pi }$) between response vectors over a set (*n* = 5000) of random selection gradients; $\varphi_{j}$ is the angle (in radians) between response vectors for **G** matrices subjected to the *j^th^* selection gradient; atan, sin, and cos denote the four-quadrant inverse tangent, sine, and cosine functions, respectively. The value of $\bar{\varphi }$ reflects matrix differences in eigenvectors, and thus will be indicative of the extent to which the compared **G**-matrices share a common orientation (with $\bar{\varphi }$ decreasing as similarity in matrix orientation increases). Further details on this method are given in Methods S3 (Supplementary Materials).

Although not having an interpretation in line with the effects of **G**-eigenstructure on response to selection, the Krzanowski geometric approach [80,81] was applied as an additional method to compare the Mainland and Island **G**-matrices in terms of orientation. By finding the closest alignment between two matrix subspaces, each defined by a subset of *k* eigenvectors within a *q*-dimensional trait space (and subject to the condition that *k* ≤ *q*/2; [80]), Krzanowski’s approach gives a bounded index of overall similarity in orientation (hereafter denoted as $\Sigma \lambda_{S}$) between the matrix subspaces being compared. This index varies from 0 to *k*, with 0 indicating that the two subspaces are dissimilar (no shared structure), whereas a value close to *k* indicates that the two subspaces are coincident (share a similar structure) (for further details on this method, see Methods S4; Supplementary Materials).

#### 2.2.2. Evolutionary Divergence among Populations

The influence of intra-specific genetic (co)variation on the evolutionary trajectory of a population and its divergence from other populations was assessed through the estimation of evolvability in the direction of evolutionary change of a population. As a complementary approach, we also did a comparison of **G** and **D** matrices in terms of size, shape, and orientation. The procedures used in this matrix comparison are described in Methods S5 (Supplementary Materials), including the estimation of phylogenetic-corrected among-population (co)variances in the **D**-matrix.

Hansen and Houle [6] suggested to evaluate the influence of genetic architecture on the direction and extent of population differentiation based on the amount of mean-scaled genetic variance available along the direction of divergence of a population. Specifically, considering the vector **z** that defines the direction of divergence of a population, observed unconditional (e(z)), and conditional (c(z)) evolvabilities in this direction are compared to the expected evolvabilities in a random direction ($\bar{e}$ and $\bar{c}$). If populations tend to diversify in directions where evolvability is much higher than expected (i.e., e(z) and c(z) greater than $\bar{e}$ and $\bar{c}$, respectively), then **G** may have shaped the direction of population differentiation (conversely, it is also possible that **G** has evolved to reflect past selection, in which case the direction of population divergence could shape **G,** and thus cause high evolvability [6]). In addition, a positive association between either e(z) or c(z) and the amount of divergence in the **z**-direction may indicate that **G** could also have affected the extent of population differentiation [9].

Table 2 provides details on the estimation of **z** as a direction of divergence of a population from the inferred ancestral states (i.e., phylogenetically-weighted trait means), as well as on the estimation of evolvabilities and the amount of divergence along a **z**-direction. The calculation of e(z) and c(z) was based on a **G** matrix that was estimated by combining all data, and thus to obtain a **G**-matrix common to all populations across the Mainland and Island groups. This followed from the **G**-matrix comparison between these groups, which indicated that the **G**-eigenstructure was reasonably well conserved at an intra-specific level (see Results). Thus, similar to other studies (e.g., [14,15]), we assumed the pooled within-population genetic (co)variances to be an approximation for describing the average of **G** over evolutionary time for each population. This pooled **G**-matrix was estimated under the model described in Equation (1), and it was mean-standardized by using estimates of phylogenetically-weighted trait means (so that **z** and **G** were on a comparable scale; Table 2). Populations were fitted as fixed effects in this model (akin to the data analyses within groups), and least-squares (phenotypic) means were estimated for each trait and population; Wald *F*-tests were used to assess the statistical significance of differences among populations for the traits, with the denominator degrees of freedom calculated as defined by Kenward and Roger [82].

#### 2.2.3. Estimation of Sampling Error for the Measures Used to Compare Matrices

Variance-covariance matrices have an estimation error and so do functions of them, such as the measures described above for matrix comparison. Assessing uncertainty in these measures followed the restricted maximum-likelihood multivariate normal (REML-MVN) sampling approach suggested by Houle and Meyer [85]. This approach samples a variance-covariance matrix that had been estimated by REML, and the matrix samples are then used to assess the sampling variability and to obtain confidence intervals for measures based on the estimated matrix. We applied REML-MVN sampling to construct 100,000 samples of either **G** or **D** matrix estimates, calculated the target measures for each sample, and generated the sampling distribution for each measure across samples. Lower and upper limits of the 95% confidence intervals for the measures of interest were approximated from the 2.5th and 97.5th percentiles, respectively, of the generated sampling distributions. Further details on the REML-MVN approach and on its application in the present study (e.g., sampling on the **L**-scale) are given in Methods S6 (Supplementary Materials).

#### 2.2.4. Evaluating the Statistical Support for Similarity of Two Estimated (Co)Variance Matrices

Evaluating the statistical support for similarity of two estimated variance-covariance matrices being compared depended on whether [19]:

. measures were computed separately for each matrix and then compared between matrices (i.e., measures that capture the potential for evolution; measures of matrix size and shape), where we generated the sampling distribution for the difference between matrices in a measure, based on 100,000 REML-MVN matrix samples drawn from either estimated matrix; or
. measures were directly calculated from a between-matrix comparison (i.e., $\bar{\varphi }$, $\Sigma \lambda_{S}$ and the angle between **g**_max_ and **d**_max_), where we computed the statistic $\Psi_{m}$—for a measure *m*, $\Psi_{m}$ evaluates whether differences within matrices due to sampling error are similar to differences between matrices (Equation (9) in [86])—and drew 100,000 pairs of REML-MVN matrix samples from either estimated matrix to generate the sampling distribution of $\Psi_{m}$.

Statistical support for similarity between estimated matrices in the evaluated measures was examined through the 95% confidence intervals approximated from the generated sampling distributions (i.e., matrix similarity was indicated by a 95% confidence interval overlapping with zero). Methods S7 (Supplementary Materials) gives further details on the procedures described above.

## 3. Results

### 3.1. Comparison of **G** Matrices for the Mainland and Island Population Groups

Table 3 presents the mean-standardized additive genetic (co)variances estimated for the **G** matrices of the Mainland and Island groups, with the variances being equivalent to the $I_{A}$ evolvabilities. Estimates of trait means, phenotypic standard deviations, and narrow-sense heritabilities are also given in Table S2 (Supplementary Materials). The $I_{A}$ estimates were highly significant (*p* < 0.001) for all traits and ranked similarly in both population groups, with the levels of $I_{A}$ being the highest for EX and the lowest for KL (Table 3). Under a model ignoring trait covariances, two-tailed LR tests conducted within a group to assess whether the $I_{A}$ estimates differed between a pair of traits indicated that either EX or KL was significantly (*p* < 0.05) different from the other three traits, whereas the difference between S/G and WD was not statistically significant (not show). Both **G** matrices tended to have negative covariances between S/G and either BD or EX, and a similar pattern of covariances of EX with the other two traits (i.e., positive with KL; low and not significant with BD) (Table 3).

In analyses jointly modeling data from both groups under Equation (1), three null hypotheses were tested to evaluate whether the **G** matrices differed in (co)variance estimates. As shown in Table 4, reduced models where both **G** matrices were constrained to have the same estimates for each of the 10 parameters, the 4 variances or the 6 covariances did not lead to a significantly worse fit (at the 5% level) than a full model where all parameters were estimated independently for each matrix. This was also observed when testing a single parameter per se, except for the genetic covariance between S/G and KL which differed significantly (*p* < 0.05) between matrices (not shown). In general, these results suggest a weak differentiation between the (co)variance patterns of the two **G**-matrices.

The results obtained for the measures reflecting the influence of **G**-eigenstructure on response to selection are given in Table 5. The Mainland-**G** had lower levels of mean evolvability ($\bar{e}$ = 0.41% and $\bar{c}$ = 0.09%) when compared to the Island-**G** ($\bar{e}$ = 0.58% and $\bar{c}$ = 0.12%), indicating that, on average, more genetic variation was available in the Island group for response to a wide range of selection gradients (Table 5). Yet, the expected ability of a multivariate phenotype to respond to any random selection vector did not appear to significantly differ between the **G** matrices. The 95% confidence interval (CI) for the difference between matrices in $\bar{e}$ (Figure S2a; Supplementary Materials) or $\bar{c}$ (Figure S2b) included zero, hence providing statistical support for matrix similarity in these measures (note that, alternatively to using evolvability differences, 95% CIs generated for ratios of $\bar{e}$ or $\bar{c}$ values overlapped with one, and thus also indicated matrix similarity). In particular, the results for $\bar{c}$ also suggested that the **G** matrices did not significantly differ in the degree to which evolvability was (on average) affected by genetic covariances between traits. This is better elucidated by the mean evolutionary autonomy over random directions ($\bar{a}$), as shown in Table 5. The **G** matrices had similar magnitudes of $\bar{a}$ (see also Figure S2c) indicating that, on average, conditional evolvabilities were 29% of the unconditional evolvabilities, which may be translated into a reasonably high level of mean evolutionary integration (*sensu* [43]) over random directions (i.e., 1 − $\bar{a}$ = 0.71) in both groups. This relative reduction in the overall level of independent evolutionary potential suggests that genetic covariances could have been important in constraining the number of directions of the phenotypic space (trait combinations) along which the focal traits would be highly evolvable. As also reflected in the magnitude of $\bar{a}$, the two **G**-matrices had an uneven distribution of the eigenvalues, with 86% of the total genetic variation being accounted for by the first eigenvector (Table S3; Supplementary Materials), indicating that both matrices had a similar ellipsoid shape (i.e., large eccentricity). Consistent with these results, the **G** matrices had similar mean flexibility ($\bar{f}$) values (Table 5; Figure S2d). The moderate magnitude of 0.6 for $\bar{f}$ indicates that, on average, a multivariate phenotype would have a limited ability to respond to (or to track with) a wide range of selection pressures, hence suggesting that genetic architecture could be important in deviating the direction of evolutionary response from the direction of selection. The evolvability, autonomy, or flexibility measures along different random directions were reasonably well correlated between the two **G**-matrices (Table S4; Supplementary Materials), providing an additional indication of matrix similarity in these measures.

**Figure 1:**
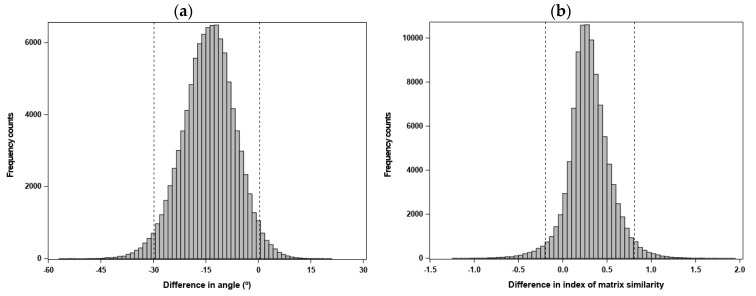
Simulated sampling distributions and 95% confidence intervals (depicted by the dashed vertical lines), obtained by the REML-MVN sampling approach [85], for the statistic $\Psi_{m}$ used to assess whether the mean-standardized **G**-matrices estimated for the Mainland and Island population groups shared a similar orientation, based on the following measures directly computed from a between-matrix comparison: (**a**) mean angle between vectors of response to a set of randomly-generated selection gradients (i.e., "random skewers"); and (**b**) Krzanowski’s index of overall similarity in orientation between matrix subspaces. For a given measure, the statistic $\Psi_{m}$ evaluates whether differences within matrices due to sampling error are similar to differences between matrices (Equation (9) in [86]; see Methods S7). When overlapping with zero, a 95% confidence interval for $\Psi_{m}$ indicates statistical support for similarity in orientation between the two matrices being compared.

The mean angle between response vectors predicted by subjecting the **G** matrices to the same selection gradients (“random skewers”) was relatively small ($\bar{\varphi }$ = 17°; Table 5), indicating that genetic variance was similarly structured in the two matrices. This shared matrix orientation was statistically supported by the 95% CI of the statistic $\Psi_{m}$ (which, for a measure *m*, compares estimates from samples of the same matrix to estimates from samples of different matrices), as shown in Figure 1a. The CI for $\Psi_{m}$ included zero (albeit marginally), indicating that the dissimilarity between matrices in $\bar{\varphi }$ was not larger than that expected from sampling error. Under the Krzanowski method, the first two eigenvectors (accounting for 96% of the total genetic variation; Table S3) of the **G** matrices defined the subspaces to be used in a two-dimensional matrix comparison. Krzanowski’s index ($\Sigma \lambda_{S}$) of overall similarity was 1.78 (95% CI: 1.32, 1.94) of a possible 2, indicating substantial trait information shared between eigenvectors of the two matrices, and thus similarity in matrix orientation. This was also supported by the 95% CI of $\Psi_{m}$, which overlapped with zero (Figure 1b). Overall, the results obtained from the comparison of the Mainland and Island **G**-matrices indicated that the **G**-eigenstructure was reasonably well conserved at an intra-specific level.

### 3.2. Evolutionary Divergence among Populations

**Figure 2:**
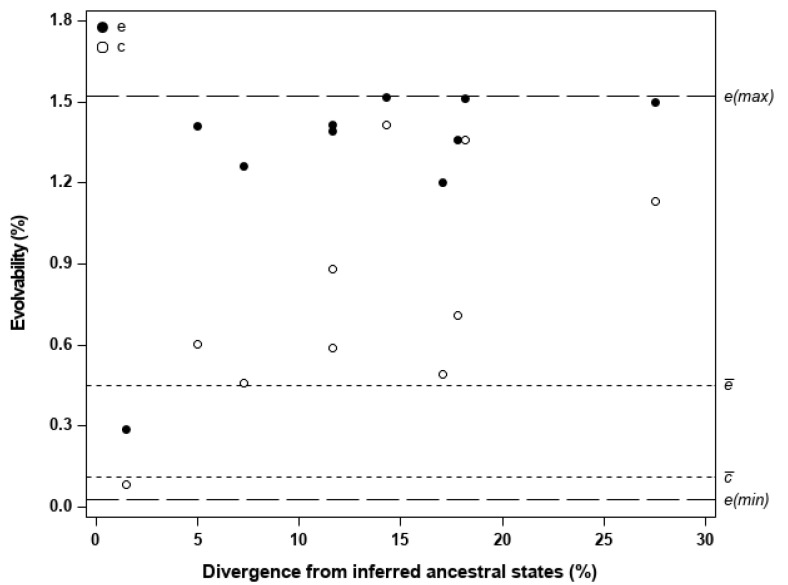
Observed mean-scaled unconditional evolvabilities (e; black dots) and conditional evolvabilities (c; white dots) for each *E. globulus* population in the direction of its divergence from the inferred ancestral states, plotted against the amount of divergence in that direction. For comparison with the observed evolvabilities along the direction of population divergence, the dashed horizontal lines indicate (after [6,9]): the expected unconditional and conditional evolvabilites in a random direction, denoted as $\bar{e}$ and $\bar{c}$, respectively; the maximum and minimum possible values for either unconditional or conditional evolvability, denoted as *e(max)* and *e(min)*, respectively. All the evolvability estimates were based on the mean-standardized **G**-matrix common to all populations, and further details are provided in Table 2.

Figure 2 shows the observed unconditional (e(z)) and conditional (c(z)) evolvabilities in the direction of divergence (**z**) of a population from the inferred ancestral states, and plotted against the amount of its divergence in this direction. The observed e(z) and c(z) were also compared to the $\bar{e}$ and $\bar{c}$, as well as to the maximum (*e(max)*) and minimum (*e(min)*) possible evolvability values, based on the mean-standardized **G** matrix common to all populations. This matrix is presented in Table S5 (Supplementary Materials); when compared to the corresponding estimates given in Table 5, the pooled **G**-matrix provided intermediate values in $\bar{e}$ or $\bar{c}$, and similar values in $\bar{a}$ or $\bar{f}$ (Table S6; Supplementary Materials). The estimated population means, used to define the **z**-vectors of evolutionary change, differed significantly (*p* < 0.001) for every trait, indicating strong population differentiation. The inferred ancestral states for the traits were: S/G = 1.94 ± 0.04; KL = 20.67% ± 0.30; BD = 546.72 kg/m^3^ ± 8.18; EX = 5.06% ± 0.36.

When the direction of population divergence is not random in regard to evolvability, the observed e(z) and c(z) will tend to deviate from $\bar{e}$ and $\bar{c}$, respectively. This was the case for the studied populations, as most of them have diversified in directions where the available mean-scaled genetic variance was substantially higher than expected in random directions. Figure 2 shows that most populations have diverged in directions where the observed e(z) were closer to *e(max)* (i.e., the evolvability along **g**_max_) than to $\bar{e}$, and also in directions where the observed c(z) were well above $\bar{c}$. The averages of the observed e(z) and c(z) were 1.287% and 0.773%, being almost three and seven times the evolvabilities expected in random directions (i.e., $\bar{e}$ = 0.452%, and $\bar{c}$ = 0.111%), respectively. This trend for populations to diversify in directions of high evolvability suggests that genetic architecture may have affected the direction of population divergence (i.e., evolutionary trajectories being biased toward the directions of major multivariate axes of genetic variation). Besides influencing the range of directions along which populations evolve, genetic architecture may also affect the extent of population divergence. The amount of population divergence along **z** tended to be positively related to c(z), with a significant linear association (*r* = 0.65, *p* = 0.041) being detected, but this trend was not evident with e(z) (Figure 2). This provides some indication that populations changing further from the inferred ancestral states had also a propensity to diverge in directions of higher evolvability, particularly when mean-scaled genetic variance along **z** was independent of trait genetic covariances in directions other than **z** (assumed to be under stabilizing selection), as reflected in c(z).

In general, the studied populations have diverged in directions closely aligned with the direction of the first eigenvector (**g**_max_) of the pooled **G**-matrix, as most of the angles between **g**_max_ and each of the **z**-vectors were relatively small and less than the critical value (i.e., 28.6° at the 5% level) from a null distribution, generated by simulating 100,000 pairs of random vectors uniformly distributed in a four-dimensional space (Table S7; Supplementary Materials). The mean angle between **g**_max_ and the **z**-vectors was 20.3°, being considerably less than expected by chance (i.e., the mean angle of 63.5° between the generated pairs of random vectors; note that these mean angles were calculated as in Equation (3)). Directions of divergence with high evolvability can be different from **g**_max_, since **G** may include other multivariate axes of higher than average genetic variance beyond **g**_max_. This was not the case here, as genetic variance was concentrated (i.e., 84% of the total) along the first eigenvector of the pooled **G**-matrix (Tables S8 and S9; Supplementary Materials), which thus may underlie the observed close alignment between **z** and **g**_max_ in most of the populations.

The main results obtained by comparing the **G** matrix common to all populations with the phylogenetically-corrected **D** matrix are summarized here, and further details are given in Supplementary Materials (Results S1). The descriptor of matrix shape provided 94% and 84% for the percentage of the total variance being accounted for by the first eigenvector of **D** and **G**, respectively (Table S9). Similarity in matrix shape was not statistically supported, as the 95% CI for the difference between matrices in this measure did not include zero (Figure S3b; Supplementary Materials), and thus indicated that **G** and **D** were not proportional. This suggests that genetic drift may not have played an important role in generating population differentiation, as **G** and **D** are expected to be proportional under neutral divergence (see Discussion). A high level of shared orientation between **G** and **D** was indicated by Krzanowski’s index of overall similarity ($\Sigma \lambda_{S}$), and by the angle between **g**_max_ and **d**_max_ (i.e., the leading axis of divergence among populations). Under a two-dimensional matrix comparison, the index $\Sigma \lambda_{S}$ was 1.91 (95% CI: 1.66, 1.98) of a possible 2 (Table S9). The **g**_max_ and **d**_max_ directions were closely aligned, with an angle of 5.9° (95% CI: 2.6°, 11.8°) between them. Statistical support for similarity in matrix orientation could not be rejected for either of these two measures (Figure S3c,d). These results suggest that axes of greatest within-population genetic (co)variance may have determined trajectories of evolutionary change, with the observed population divergence occurring mainly close to the direction of a genetic line of least resistance.

## 4. Discussion

We aimed to assess the extent to which the **G**-eigenstructure for four wood properties was conserved among populations of *E. globulus*, and evaluate its influence on the evolutionary divergence among populations. Given the radiation of related eucalypts in southern Australia, this population divergence would have occurred within the last 2–3 M years, when accelerated radiation of the southern clades of eucalypts occurred with the onset of increasing aridity in Australia (Figure S8 in [87]). Genome-wide phylogenetic studies indicate that *E. pseudoglobulus* is the sister species to *E. globulus* (Figure 9 in [88]). The Mainland populations of *E. globulus* have closer affinities to this sister species than do the Tasmanian populations [62], suggesting more recent divergence of the Island populations. This divergence most likely occurred over the last 0.5 M years, over a period when the cycle of glaciations would have produced multiple opportunities for migration between the mainland and the islands of Tasmania due to lowered sea levels in glacial periods creating a land-bridge, followed by interglacial isolation with the formation of Bass Strait [65,89]. The eastern side of the land-bridge is more persistent due to shallower seas, where the Furneaux islands and Tasmania (which comprise the Island group in the present study) would have been the last connection to be severed as sea-levels rose [90]. While the main contemporary distribution of *E. globulus* is in eastern and south-eastern Tasmania, studies of the maternally inherited chloroplast DNA suggest that *E. globulus* colonized Tasmania not via the eastern land-bridge but via a wetter western route from the Otways region, where the species now only occurs as a series of small disjunct remnants [91]. While the timing of the colonization is unknown, there have been four major glacial periods over the last 0.5 M years [89] when this would have been possible, the longest ca. 405 to 330 K years ago, and the most recent during the Last Glacial Maximum (22–18 K years ago) with the land-bridge flooded ca. 12 K years ago [92]. Given that tall forest eucalypts in Tasmania may reach 500 years of age [93], with generation times of between 200 and 500 years, the Mainland and Island populations would have been most recently spatially isolated for anywhere between 24 to 60 generations. Regardless of the timing of colonization and extent of isolation, the observed population differentiation would have been associated with latitudinal variation in climate superimposed on dramatic temporal changes through the glacial transition(s) [92].

For conspecific populations derived from a common ancestor and experiencing similar genetic constraints, Schluter’s [7] hypothesis predicts that divergence in multivariate mean phenotype among evolving populations will be biased (i.e., relative to the direction of selection) toward directions of greatest genetic variance of the **G** matrix in the ancestral population. For a set of traits, the predictive importance of this hypothesis will depend on whether recent selection mirrors historical selection, and on patterns of genetic (co)variance remaining fairly stable over evolutionary time (and thus be reflected in measured **G**-matrices of each of the diverging populations). These assumptions could be plausible over moderate time frames as adaptation may take a while following the introduction of populations into new habitats [40]. Empirical studies often find that axes of genetic variance are well preserved across conspecific populations or closely-related species [24,25], even in face of differences in natural selection [32], and after 20–40 millions of years of divergence [18]. Thus, even under an evolving **G**, the structure of genetic (co)variances within populations may remain (at least partly) conserved over evolutionary time. In the present case, given the tree life cycle, population divergence times (potentially less than 500 K years) of the *E. globulus* populations would be moderate. For the focal wood properties, there appears to have been a weak differentiation in **G**-eigenstructure following the colonization of Tasmania, as the **G**-matrices of the Mainland and Island groups showed similarity in average measures that capture the expected potential for evolution over a wide range of selection gradients on the traits. The weak differentiation observed in **G**-eigenstructure could suggest that the genetic architecture for the studied traits may have been relatively well kept through time. However, it could also reflect that **G** may have evolved in a consistent manner to become aligned with the adaptive landscape, so that natural selection has shaped similarly genetic (co)variation within populations ([25]; see discussion below). Yet, while it may be the result of various interacting mechanisms (see the Introduction for references of simulation studies), the observed **G**-conservatism justified the pooling of **G**-matrices across the population groups to approach an estimate of the average of **G** over time, which could then be used in evolutionary inference for the timescale of interest (e.g., [11,12,14,15,94]).

The observed **G**-conservatism may also support a putative enduring influence of genetic constraints in causing deviations of populations from optimal response to selection over evolutionary time [19]. Consistent with this possibility, the present study found that most populations diverged in directions of high evolvability, and also that populations diverging in directions of higher conditional evolvability tended to change further from the inferred ancestral states. A strong relationship between **G** and divergence is likely for traits with a high degree of evolutionary integration [17], as also indicated by the relatively low value of the average autonomy (i.e., $\bar{a}$ ≈ 0.3) obtained for the wood properties. The distribution of the additive genetic variance was uneven in the estimated **G** for the studied traits, with an expected limited ability for response to a broad range of selection gradients (i.e., $\bar{f}$ ≈ 0.6) reflecting the large percentage (i.e., >80% of the total) of the variance concentrated on the **g**_max_ direction, and thus indicating a possible role of genetic constraints on population differentiation (e.g., [11,12,13,14]). Indeed, nearly all populations diverged from the inferred ancestral states in a direction that was not significantly different from the genetic line of least resistance (Table S7), a trend that was supported by the strong alignment (i.e., 5.9°; Table S9) between the first eigenvectors of the **G** matrix common to all populations and the phylogenetically-corrected **D** matrix. This suggests that population trajectories may have been biased toward a direction determined by genetic (co)variation in wood extractives (EX) (which was the trait with the greatest $I_{A}$ evolvability), lignin content (KL) and S/G (Tables S5 and S8). There is an indication that the focal traits may be genetically integrated to some degree, based on the co-location of quantitative-trait loci (QTL) detected within four *E. globulus* full sib-families [95]. However, as suggested in empirical studies [17,18], the effects on standing genetic (co)variation patterns produced by the per-generation input of new mutations are likely to have an important contribution to long-term genetic constraints imposed on phenotypic evolution. In particular, Houle et al. [96] demonstrated a strong positive relationship between variation produced by mutation, standing genetic variation, and the rate of evolution over a period of 40 million years in *Drosophila melanogaster*. Nevertheless, mechanisms other than persistent genetic constraints may also have contributed to the association we have observed between genetic architecture and population divergence, as discussed below.

As indicated in two recent empirical studies [18,37], and noted earlier [7], the relationship between genetic architecture and population divergence may arise because both are shaped by the same natural selection pressures. This entails that **G** evolves to become aligned with the adaptive landscape, so that natural selection shapes both **G** and **D** in a similar direction [25]. Such a scenario could be plausible for functionally integrated traits with strong adaptive correlations, where correlational selection within populations would lead to a tendency for selective differences among populations to occur along an adaptive ridge in the phenotypic space (i.e., directions of population divergence in close alignment with **ω**_max_; [23]). Correlational selection for co-adaptation may be a possibility for the studied traits, given the relationship between their functions (as outlined in the Introduction) and also as suggested by the pattern of strong correlated evolution among populations (described by the covariances in the **D**-matrix; Table S5). In particular, correlational selection for resistance to wood decay could be one possibility, as: (i) there are likely local topographic (gully versus slopes [97]) and broad-scale [98] gradients in disease risk across the range of *E. globulus*; and (ii) the trait relationships on the first eigenvector of the **G** and **D** matrices (Table S8) are consistent with evolutionary changes expected to increase resistance to wood decay (i.e., increasing EX, KL and, depending upon the type of decay, decreasing S/G; see Introduction). Theoretical work developed by Jones et al. [10,26] indicated that conditions inducing high orientation stability of **G**—i.e., the alignment of an adaptive landscape producing strong correlational selection and a **M**-matrix describing strong mutational covariance, coupled with large population size—did also promote the evolution of an eccentric **G**-matrix (see also [29]), as may have occurred in the present case. Directional selection can confer greater orientation stability on **G** relative to stabilizing selection alone and, in combination with an alignment of the adaptive landscape and **M**, a steady movement of the selective optimum along **ω**_max_ facilitates orientation stability of **G** by reinforcing the evolution of an eccentric **G**-matrix [25,26]. An evolving **M**-matrix was also found to further enhance orientation stability of **G** relative to a static mutational architecture, especially as **M** evolves toward alignment with the adaptive landscape [27,28]. This triple alignment of **G**, **M**, and the adaptive landscape could be produced by a landscape configuration that supports persistent correlational (and stabilizing) selection within populations over evolutionary time [25], and such a coordinated pattern of genetic variation, mutation and selection could have contributed to the observed similarity of **G** matrices, as well as to the observed alignment of **G** and **D** matrices.

Migration is another factor which could lead to the observed alignment between genetic architecture and population divergence [7]. Using a mainland-island migration model, Guillaume and Whitlock [30] indicated that continuous high levels of gene flow from the mainland population could affect all features (size, shape, and orientation) describing an island **G**-eigenstructure, and lead to an increase in genetic variance along the direction of population divergence. However, the changes caused by migration were found to be slow and to increase over time, and tended to be reduced with increased correlational selection and/or mutation correlation [30]. In the present case, the contribution of gene flow cannot be completely dismissed, considering: (i) *E. globulus* to be pollinated by animals, including a key bird pollinator which annually migrates between the Mainland and Island populations [67,99]; and (ii) the demonstrated potential for post-dispersal selection favoring the products of long-distance dispersal due to a release from inbreeding depression [100]. While the effective pollen dispersal curve has been shown to be fat-tailed, the average dispersal distance has been estimated as only between 69 m and 833 m, depending on the studied population [74]. Nevertheless, gene flow would be expected between more-or-less continuous populations within either region, but it is unlikely to be strong between Mainland and Island groups which are separated by a disjunction of over 130 km. While the influence of inter-population migration cannot be completely discounted, it is unlikely in the present case as a continuous high level of gene flow is required to have a major effect on the **G**-eigenstructure, as well as in inducing an alignment between genetic (co)variation and population divergence [30].

Another important issue is whether the observed alignment of genetic architecture and population divergence is caused by genetic drift. Phillips et al. [101] showed that genetic drift can cause among-population variation in **G** by influencing all features describing the **G**-eigenstructure within individual populations but, on average, it will lead to proportionality of **G** and **D**. Thus, when population differentiation is due to genetic drift alone, **G** and **D** will have a similar expectation in terms of shape, but also in orientation so that population means will diverge along directions of greatest genetic variance [2,39,101,102]. In this sense, proportionality of **G** and **D** matrices reflecting matrix similarity in both shape and orientation could be indicative of genetic architecture biasing population divergence that is generated by drift [39]. However, in the present case, we would argue against an over-riding role of drift in generating population differentiation on the basis of: (i) while **G** and **D** did not differ significantly in size and orientation, they did in shape (i.e., they were not proportional; Table S9, Figure S3); and (ii) evidence for divergent selection acting on all of the studied wood properties (i.e., statistically significant *Q_ST_* > *F_ST_* [60]). While drift can alter the course of evolution via its continuing effects on **G**, it is unlikely to have played a major role in the evolutionary history of populations when comparative studies indicate that **G** has remained relatively stable over long periods of time [103], as suggested from the Mainland-Island comparison in the present case. Certainly with a minimum of 24 to 60 generations since the spatial isolation of the Mainland and Island population groups, the role of genetic drift is unlikely, especially with the populations sampled coming from large, more-or-less continuous, regional distributions of the species, and molecular studies revealing no signs of inbreeding or loss of genetic diversity which would signal sustained population bottlenecks [62,66].

Finally, the studied populations were evaluated in a common-garden experiment and not in their natural habitats. Environmentally-induced variation in **G** has been found to be on the same order of magnitude as observed differences in **G** among species (e.g., [104]) or conspecific populations (e.g., [36]). Therefore, it is possible that deviations of the common-garden conditions from the natural environments of the populations may have affected trait (co)variation at both the within- and among-population levels, as a result of genotype-by-environment interaction. However, multiple common-garden experiments of *E. globulus* have shown that wood properties exhibit relatively low genotype-by-environment interaction, particularly when compared to growth traits [95,105,106].

## 5. Conclusions

The present study indicated that population divergence for the set of wood properties occurred along a genetic line of least resistance. While we did not have sufficient statistical power to warrant testing **G**-matrix variation among individual populations within the Mainland and Island groups, there was no evidence for a systematic difference in the **G** matrices of the two groups as would be expected if natural selection, gene flow or genetic drift during the Island colonization differentially impacted **G**-matrix evolution. In general, the unconditional evolvability estimates suggested that standing additive genetic variance would not substantially limit the traits to respond to directional selection (but see lignin). However, as indicated by the estimated average level of autonomy of this genetic variance, the reasonably high degree of evolutionary integration of the focal traits may have been important in underlying the observed close relationship between genetic architecture and population trajectories. It is not possible to ascertain whether these results reflect genetic constraints, or an association between evolutionary forces that may have shaped genetic variation and those that have led to population diversification. The latter scenario could arise from genetic (co)variance patterns that may have consistently evolved to become aligned with natural selection, since genetic drift and/or migration may not have played an important role in generating divergence among the studied populations. Yet, as the **G**-eigenstructure appeared to be relatively well preserved at an intra-specific level, and given the moderate evolutionary timescale involved, the potential effects of genetic constraints in influencing the evolution and diversification of the *E. globulus* populations for the set of focal traits may not be discounted.

## Figures and Tables

**Table 1:** Multivariate measures capturing the potential for evolution that were evaluated to compare the Mainland and Island **G**-matrices.

Symbol	Measure	Interpretation	Results
β	A *q* × 1 (*q* = number of traits) vector, where its elements were randomly drawn from a normal distribution with a mean of 0 and a variance of 1; each vector was then normalized to unit length.	Selection gradient, representing directional selection acting on each trait.	n/a
e(β)	Unconditional evolvability along β, $\mathrm{calculated}\;\mathrm{by}\;\beta^{\prime }G\beta$, where G is the mean-standardized additive genetic variance-covariance matrix for either the Mainland or Island groups; ’ denotes the transpose operator.	Ability of a multivariate phenotype to change along β, without regard for the presence of constraints reflected in **G**-matrix structure.	n/a
c(β)	Conditional evolvability along β, $\mathrm{calculated}\;\mathrm{by}\;{\left(\beta^{\prime }G^{-1}\beta \right)}^{-1}$, where G is defined as above; ’ and ^−1^ denote the transpose and the inverse operators, respectively.	Ability of a multivariate phenotype to change along β independently of the variation in the remaining (orthogonal) directions of the phenotypic space, which are assumed to be under stabilizing selection and in equilibrium with β, so that no response is allowed along any direction other than β. The c(β)intends to quantify the effects of genetic constraints on evolvability: c(β)is typically lower than e(β), and they will tend to approach when variation along β is genetically uncorrelated with variation along the directions orthogonal to β.	n/a
a(β)	Autonomy along β, calculated by c(β)/e(β).	Proportion of evolvability along β that is left after reduction due to genetic covariances with trait combinations in the orthogonal directions to β, assumed to be under stabilizing selection.	n/a
*f*(β)	Flexibility along β, $\mathrm{calculated}\;\mathrm{by}\;\cos(\beta ,\;\Delta \bar{Z})$, which is the cosine of the angle between a β$\;\mathrm{vector}\;\mathrm{and}\;a\;\mathrm{vector}\;\Delta \bar{Z}\;=\;G\beta$ of predicted responses to selection for the traits.	Extent to which a **G**-matrix deflects the response vector from the direction of the selection gradient vector, and thus reflecting the ability of a population to track with the direction of selection (i.e. a more “flexible” population tracks closer to the direction of selection).	n/a
$\bar{e}$	Mean unconditional evolvability, $\mathrm{calculated}\;\mathrm{by}\;E\left[\beta^{\prime }G\beta \right]$.	$\mathrm{Expected}\;\mathrm{unconditional}\;\mathrm{evolvability}\;\mathrm{in}\;a\;\mathrm{random}\;\mathrm{direction}.\;\mathrm{The}\;\bar{e}$ is proportional to **G**-matrix size.	Table 5 Figure S2a
$\bar{c}$	Mean conditional evolvability, $\mathrm{calculated}\;\mathrm{by}\;E\left[{\left(\beta^{\prime }G^{-1}\beta \right)}^{-1}\right]$.	$\mathrm{Expected}\;\mathrm{conditional}\;\mathrm{evolvability}\;\mathrm{in}\;a\;\mathrm{random}\;\mathrm{direction}.\;\mathrm{The}\;\bar{c}$ is proportional to **G**-matrix size and, by accounting for trait genetic covariances, it will also reflect **G**-matrix shape.	Table 5 Figure S2b
$\bar{a}$	Mean autonomy, calculated by E[c(β)/e(β)].	$\mathrm{Overall}\;\mathrm{autonomy}\;\mathrm{averaged}\;\mathrm{over}\;\mathrm{random}\;\mathrm{selection}\;\mathrm{directions},\;\mathrm{and}\;\mathrm{used}\;\mathrm{to}\;\mathrm{capture}\;\mathrm{the}\;\mathrm{degree}\;\mathrm{of}\;\mathrm{evolutionary}\;\mathrm{constraint}\;\mathrm{inherent}\;\mathrm{in}\;a\;G\;\mathrm{matrix}.\;\mathrm{The}\;\bar{a}$ will decrease with increasing variation among the eigenvalues of **G** (indicating that the quantitative effects of genetic constraints may be stronger), and it will have a value of one only if all traits are genetically uncorrelated and have the same genetic variance.	Table 5 Figure S2c
$\bar{f}$	Mean flexibility, $\mathrm{calculated}\;\mathrm{by}\;E[\cos(\beta ,\;\Delta \bar{Z})]$.	$\mathrm{Overall}\;\mathrm{flexibility}\;\mathrm{averaged}\;\mathrm{over}\;\mathrm{random}\;\mathrm{selection}\;\mathrm{directions}.\;A\;\mathrm{value}\;\mathrm{of}\;\bar{f}$$\;\mathrm{approaching}\;\mathrm{one}\;\mathrm{will}\;\mathrm{indicate}\;\mathrm{that},\;\mathrm{on}\;\mathrm{average},\;\mathrm{the}\;\mathrm{directions}\;\mathrm{of}\;\mathrm{response}\;\mathrm{and}\;\mathrm{selection}\;\mathrm{are}\;\mathrm{well}\;\mathrm{aligned}\;\mathrm{for}\;a\;\mathrm{wide}\;\mathrm{range}\;\mathrm{of}\;\mathrm{selection}\;\mathrm{vectors},\;\mathrm{and}\;\mathrm{thus}\;\mathrm{the}\;\mathrm{direction}\;\mathrm{of}\;\mathrm{response}\;\mathrm{to}\;\mathrm{selection}\;\mathrm{is}\;\mathrm{expected}\;\mathrm{to}\;\mathrm{be}\;\mathrm{less}\;\mathrm{biased}\;\mathrm{by}\;G;\;a\;\mathrm{strong}\;\mathrm{negative}\;\mathrm{linear}\;\mathrm{relationship}\;\mathrm{between}\;\bar{f}$ and matrix eccentricity has been reported [11,12,78].	Table 5 Figure S2d

The $\bar{e}$, $\bar{c}$, $\bar{a}$, and $\bar{f}$ represent average values over 5000 random selection gradients uniformly distributed in the *q*-dimensional space, and generated as described above for β. The evolvability and autonomy measures are based on Hansen and Houle [6], and flexibility is based on Marroig et al. [78]. n/a = not available.

**Table 2:** Measures used to assess the influence of intra-specific genetic (co)variation on the evolutionary trajectory of a population and its divergence from other populations.

Symbol	Measure	Interpretation	Results
**z**	A *q* × 1 (*q* = number of traits) divergence vector, obtained for each population by (e.g., [11]): (i) calculating the difference between a vector of phenotypic (least-squares) trait means for the population and a vector of phylogenetically- weighted means estimated for each trait over populations; (ii) mean-standardizing the difference vector via the phylogenetically-weighted trait means; and (iii) normalizing the mean-standardized difference vector to a unit length. Based on the reconstructed phylogeny of the studied *E. globulus* populations (Methods S1; Figure S1), the vector of phylogenetically-weighted means was estimated by using the *fastAnc* function in the R-package Phytools developed by Revell [83], assuming a Brownian motion model of trait evolution. This vector corresponds to the inferred ancestral states for each trait at the root node of the phylogenetic tree, under the assumed model of trait evolution [84].	Direction of divergence of a population from the inferred ancestral states.	n/a
e(z)	Unconditional evolvability along **z**, $\mathrm{calculated}\;\mathrm{by}\;z^{\prime }Gz$, where G is the mean-standardized additive genetic variance-covariance matrix common to all populations; ’ denotes the transpose operator.	Ability of a population to change along z, without regard for the presence of constraints reflected in G−matrix structure. Thus, the e(z) measures the mean-scaled genetic variance available for a population to diverge along **z**, without accounting for genetic covariances with trait combinations in directions other than **z**.	Figure 2 Table S7
c(z)	Conditional evolvability along **z**, $\mathrm{calculated}\;\mathrm{by}\;{\left(z^{\prime }G^{-1}z\right)}^{-1}$, where G is defined as above; ’ and ^−1^ denote the transpose and the inverse operators, respectively.	Ability of a population to change along z independently of the variation in the remaining (orthogonal) directions of the phenotypic space, which are assumed to be under stabilizing selection and in equilibrium with z, so that no response is allowed along any direction other than z. Thus, the c(z) measures the mean-scaled genetic variance available for a population to diverge along **z**, assuming that traits are constrained to evolve only in this direction.	Figure 2 Table S7
$\bar{e}$	Mean unconditional evolvability, $\mathrm{calculated}\;\mathrm{by}\;E\left[\beta^{\prime }G\beta \right]$.	Expected unconditional evolvability in a random direction.	Figure 2 Table S6
$\bar{c}$	Mean conditional evolvability, $\mathrm{calculated}\;\mathrm{by}\;E\left[{\left(\beta^{\prime }G^{-1}\beta \right)}^{-1}\right]$.	Expected conditional evolvability in a random direction.	Figure 2 Table S6
*e(max)*	Maximum possible evolvability, given by the eigenvalue of the first eigenvector of **G**.	Mean-scaled genetic variance available along the direction of the first eigenvector of **G**.	Figure 2 Table S8
*e(min)*	Minimum possible evolvability, given by the eigenvalue of the last eigenvector of **G**.	Mean-scaled genetic variance available along the direction of the last eigenvector of **G**.	Figure 2 Table S8
Amount of divergence	Amount of divergence in the z-direction, measured by the Euclidean norm of the mean-standardized difference vector [defined as in (ii) above].	Extent to which a population has diverged from the inferred ancestral states in the **z**-direction.	Figure 2 Table S7

The $\bar{e}$ and $\bar{c}$ represent average values over 5000 random selection gradients uniformly distributed in the *q*-dimensional space, and generated as described in Table 1 for β. The evolvability measures are based on Hansen and Houle [6], and Hansen and Voje [9]. n/a = not available.

**Table 3:** Additive genetic (**G**) variance-covariance matrices estimated for wood traits (S/G, KL, BD, and EX) within the Mainland and Island population groups of *E. globulus*. Parameter estimates are given together with their standard errors for variances (diagonal), covariances (below diagonal), and correlations (above diagonal).

	S/G	KL	BD	EX
Mainland **G**-matrix				
S/G	0.160 ± 0.037 (*p* < 0.001)	−0.66 ± 0.14 (*p* < 0.001)	−0.23 ± 0.15 (*p* > 0.05) ^(a)^	−0.80 ± 0.13 (*p* < 0.001)
KL	−0.062 ± 0.020 (*p* < 0.001)	0.056 ± 0.017 (*p* < 0.001)	0.04 ± 0.18 (*p* > 0.05)	0.78 ± 0.11 (*p* = 0.002)
BD	−0.036 ± 0.026 (*p* > 0.05)	0.004 ± 0.017 (*p* > 0.05)	0.157 ± 0.034 (*p* < 0.001)	0.17 ± 0.19 (*p* > 0.05)
EX	−0.361 ± 0.106 (*p* < 0.001)	0.208 ± 0.078 (*p* = 0.002)	0.074 ± 0.090 (*p* > 0.05)	1.261 ± 0.460 (*p* < 0.001)
Island **G**-matrix				
S/G	0.131 ± 0.029 (*p* < 0.001)	0.05 ± 0.18 (*p* > 0.05)	−0.38 ± 0.14 (*p* = 0.009)	−0.40 ± 0.14 (*p* = 0.012)
KL	0.005 ± 0.016 (*p* > 0.05)	0.059 ± 0.018 (*p* < 0.001)	−0.38 ± 0.15 (*p* = 0.026)	0.60 ± 0.12 (*p* = 0.001)
BD	−0.058 ± 0.023 (*p* = 0.009)	−0.039 ± 0.019 (*p* = 0.026)	0.176 ± 0.035 (*p* < 0.001)	−0.13 ± 0.15 (*p* > 0.05)
EX	−0.201 ± 0.085 (*p* = 0.012)	0.206 ± 0.073 (*p* = 0.001)	−0.074 ± 0.089 (*p* > 0.05)	1.972 ± 0.448 (*p* < 0.001)

Traits: S/G = syringyl to guaiacyl ratio; KL = lignin (Klason) content; BD = basic density; EX = extractive content. All the (co)variance estimates presented in the table are multiplied by 100, and pertain to mean-standardized matrices. The variance estimates in the diagonals can be interpreted as univariate mean-scaled evolvabilities. Significance probabilities from likelihood-ratio tests are given within parentheses (note that testing a variance estimate was pursued by fitting a univariate model that ignored the trait covariances, in order to avoid estimation and convergence problems that could arise with a multivariate model when constraining a variance estimate to remain fixed at zero under the null hypothesis). ^(a)^ *p* ≈ 0.10.

**Table 4:** Results from two-tailed likelihood-ratio (LR) tests that were applied to test whether estimates of additive genetic (co)variances differed significantly between the additive genetic (**G**) variance-covariance matrices, estimated within the Mainland and Island population groups of *E. globulus*.

Null Hypothesis	LR Test Statistic	Degrees of Freedom ^(a)^	*p*-Value
1: Variances for a trait and covariances between a pair of traits do not differ amongst the two groups	17.16	10	0.071
2: Variances for a trait do not differ amongst the two groups	2.26	4	0.688
3: Covariances between a pair of traits do not differ amongst the two groups ^(b)^	12.02	6	0.062

Both of the **G**-matrices were mean-standardized. ^(a)^ The number of total (co)variance parameters fitted in the full (unconstrained) model was 60, as opposed to 50, 56, and 54 parameters fitted in the reduced (constrained) models under the null hypotheses 1, 2, and 3, respectively. ^(b)^ Testing differences amongst the two groups in genetic correlations, rather than in genetic covariances, led to similar conclusions: LR test statistic = 10.86, *p*-value = 0.093 (6 degrees of freedom).

**Table 5:** Comparison of the additive genetic (**G**) variance-covariance matrices, estimated within the Mainland and Island population groups of *E. globulus*, based on measures reflecting the influence of **G**-eigenstructure on response to selection (with 95% confidence intervals within parentheses).

	$\bar{e}$	$\bar{c}$	$\bar{a}$	$\bar{f}$	$\bar{\varphi }$
Mainland **G**-matrix	0.409 (0.263, 0.667)	0.085 (0.039, 0.119)	0.290 (0.138, 0.375)	0.606 (0.547, 0.676)	17.0 (10.0, 27.6)
Island **G**-matrix	0.581 (0.420, 0.860)	0.118 (0.076, 0.156)	0.286 (0.174, 0.364)	0.602 (0.549, 0.656)	

Mean values of unconditional evolvability ($\bar{e}$), conditional evolvability ($\bar{c}$), autonomy ($\bar{a}$), and flexibility ($\bar{f}$) were calculated separately for each matrix, whereas the mean angle ($\bar{\varphi }$, in degrees) between vectors of response to a set of randomly-generated selection gradients (i.e., “random skewers”) was directly computed from a between-matrix comparison. Both of the **G**-matrices were mean-standardized. See Figure 1a and Figure S2 to evaluate the statistical support for similarity of the **G** matrices in the measures provided in the table. The $\bar{e}$ and $\bar{c}$ values are multiplied by 100.

## Supplementary Materials

The following are available online at www.mdpi.com/xxx/s1/, Methods S1: Reconstruction of the phylogeny of the *E. globulus* populations. Methods S2: Measures that capture the potential for evolution. Methods S3: Matrix comparison using the “random skewers” method. Methods S4: Krzanowski’s geometric approach. Methods S5: Comparison of **G** and **D** matrices. Methods S6: REML-MVN sampling approach. Methods S7: Statistical support for similarity of two estimated (co)variance matrices. Results S1: Comparison of **G** and **D** matrices. Figure S1: The phylogenetic tree generated for the ten studied populations of *E. globulus*. Figure S2: Differences between the Mainland and Island **G**-matrices in measures that capture the potential for evolution. Figure S3: Differences between a **G** matrix common to all populations and a phylogenetically-corrected **D** matrix in measures of matrix size, shape and orientation. Table S1: Identification of the studied populations within the Mainland and Island groups, and number of families and trees measured per population. Table S2: Means, phenotypic standard deviations, mean-scaled within-family variance, and narrow-sense heritabilities for the measured wood property traits. Table S3: Principal component analysis of the **G** matrices estimated within the Mainland and Island groups. Table S4: Pearson correlations between the estimated **G**-matrices of the Mainland and Island groups for measures that capture the potential for evolution along different random directions. Table S5: **G** and **D** matrices estimated across all the studied populations. Table S6: Mean values of unconditional evolvability, conditional evolvability, autonomy and flexibility based on the estimated **G**-matrix common to all populations. Table S7: Amount of divergence from the inferred ancestral states, unconditional and conditional evolvabilities along the direction of divergence (**z**), and angle between **z** and **g**_max_, for the studied populations. Table S8: Principal component analysis of the **G** and **D** matrices estimated across all the studied populations. Table S9: Comparison of the **G** and **D** matrices estimated across all the studied populations in measures of matrix size, shape, and orientation.
