# Supplementary material for: Population Divergence along a Genetic Line of Least Resistance in the Tree Species Eucalyptus globulus
Source: Genes (Basel). 2020 Sep 18;11(9):1095. doi: 10.3390/genes11091095 (PMC7565133; doi:10.3390/genes11091095)
Supplement: Supplementary file 1 [file genes-11-01095-s001.pdf]

Supplementary Materials

Contents

1. Supplementary methods

Methods S1 (page 2)
Methods S2 (pages 3 to 5)
Methods S3 (page 6)
Methods S4 (pages 6 to 8)
Methods S5 (pages 8 to 10)
Methods S6 (pages 10 to 11)
Methods S7 (pages 12 to 13)

2. Supplementary results

Results S1 (pages 14 to 15)

3. Supplementary figures

Figure S1 (page 16)
Figure S2 (page 17)
Figure S3 (page 18)

4. Supplementary tables

Table S1 (page 19)
Table S2 (page 20)
Table S3 (page 21)
Table S4 (page 22)
Table S5 (page 23)
Table S6 (page 24)
Table S7 (page 25)
Table S8 (page 26)
Table S9 (page 27)

5. Supplementary references (pages 28 to 31)

1. Supplementary methods

Methods S1: Reconstruction of the phylogeny of the *E. globulus* populations

The phylogeny of the *E. globulus* populations was reconstructed using a set of 812,158 high-quality, genome-wide SNPs called from whole-genome shotgun sequence data (Illumina Paired-read) obtained from 136 individuals in 17 *E. globulus* populations. The SNPs were filtered by Jacob Butler (University of Tasmania) from an original SNP data set generated by Josquin Tibbits (AgriBioc) and Hossein Kahrood (University of Melbourne). The trees sampled were from the field trial studied in the current work, with each individual belonging to a unique open-pollinated family [1]. Nei's genetic distances [2] among populations were calculated using the StAMPP R-package [3], and the matrix of genetic distances was used to calculate a phylogenetic tree via the neighbour-joining method (e.g. [4]). *E. globulus* belongs to a complex of four taxa (*E. maideni*, *E. bicostata*, *E. peudoglobulus* and *E. globulus*) which have geographically and morphologically distinctive cores, but clinally integrade [5]. Genome-wide phylogenetic studies indicate that *E. peudoglobulus* is the sister species to *E. globulus* (Figure 9 in [6]). The mainland populations of *E. globulus*, particularly the Strezlecki Ranges population, have closer affinities to this sister species than do the Tasmanian and Bass Strait island populations [5]. Accordingly, the neighbour-joining tree was rooted on the Strezlecki population, and trimmed to the ten populations used in the present study. Assuming equal rates of evolutionary change for the ten populations, the branches of this tree were then extended to produce an ultrametric tree (Figure S1). Phylogenetic analyses were undertaken using the phytools R-package [7].

Methods S2: Measures that capture the potential for evolution

The mean-standardized **G** matrices from the Mainland and Island groups were compared by using multivariate measures that capture the potential for evolution, as developed by Hansen and Houle [8] (see also [9]). In this context, we evaluated the unconditional evolvability [$e(\boldsymbol{\beta})$], the conditional evolvability [$c(\boldsymbol{\beta})$] and the evolutionary autonomy [$a(\boldsymbol{\beta})$], which can be computed given a **G** matrix and a vector $\boldsymbol{\beta}$ of selection gradients (comprising directional selection acting on a trait). For a vector $\boldsymbol{\beta}$ normalized to unit length: $e(\boldsymbol{\beta})$ is estimated from the length of the projection of the response vector ($\Delta\bar{\mathbf{z}} = \mathbf{G}\boldsymbol{\beta}$) onto $\boldsymbol{\beta}$, and computed as $\boldsymbol{\beta}'\mathbf{G}\boldsymbol{\beta}$, where ' denotes the transpose operator; $c(\boldsymbol{\beta})$ is estimated by $(\boldsymbol{\beta}'\mathbf{G}^{-1}\boldsymbol{\beta})^{-1}$, where $^{-1}$ denotes the inverse operator and **G** is assumed to be positive definite; and $a(\boldsymbol{\beta})$ is calculated by the ratio $c(\boldsymbol{\beta})/e(\boldsymbol{\beta})$ [8]. In the absence of knowledge about the actual $\boldsymbol{\beta}$ vector operating on the traits, we computed these multivariate measures averaged over a large number of $\boldsymbol{\beta}$ vectors, that were randomly sampled within a wide range of directions of the phenotypic space and then normalized to unit length (see the Materials and Methods, and Table 1).

The $e(\boldsymbol{\beta})$ measures the potential of a multivariate phenotype to respond to selection in a $\boldsymbol{\beta}$ direction, without regard for the presence of constraints reflected in **G**-matrix structure. The $e(\boldsymbol{\beta})$ calculated on the original trait scale will not be a sensible measure of evolutionary potential unless the traits are measured on comparable scales. For mean-standardized **G** and $\boldsymbol{\beta}$, the $e(\boldsymbol{\beta})$ has a readily interpretation: it corresponds to the expected proportional change per generation in a mean-standardized trait vector when the directional selection along $\boldsymbol{\beta}$ is of unit strength (i.e. assuming the strength of selection on the trait vector to be as strong as that on relative fitness regarded as a trait; [8]). When placed in the univariate context, this interpretation implies that the evolvability of a single trait (denote as I_A in the present article) will equal its mean-standardized additive genetic variance [8]. This univariate version of unconditional evolvability has been

indicated by Hansen *et al.* [10] as more suitable than narrow-sense heritability for measuring the ability of a given trait to respond to selection in natural populations.

Evolutionary change in a trait or in a trait combination under directional selection may be constrained by genetic covariances with other characters that are under stabilizing selection in nature [8, 9, 11]. These genetic constraints may reduce the actual genetic variance available for directional selection to act on a trait (or a trait combination), which is not incorporated in the unconditional evolvability measure. Hansen *et al.* [11] introduced the concept of conditional evolvability to quantify evolutionary response in a trait that may be constrained by its genetic covariances with other characters, assumed to be under stabilizing selection and kept constant near their fitness optima. In this context, an equilibrium is considered to be reached between indirect selection from the focal trait, shifting the genetically correlated characters away from their optima, and direct selection on these characters moving them back toward their optima [9]. At this equilibrium state, directional selection on a focal trait may only be able to use a specific part of the total available genetic variance, as some of this variance may not be independent of the constraining effects of genetically correlated characters under stabilizing selection [11]. Extending further this concept to quantify constrained evolution along a selection gradient, Hansen and Houle [8] have proposed to estimate the multivariate conditional evolvability in a $\boldsymbol{\beta}$ direction [$c(\boldsymbol{\beta})$]. The $c(\boldsymbol{\beta})$ measures the amount of evolutionary change possible along $\boldsymbol{\beta}$ given that the response is not allowed to deviate from the direction of selection, as it may occur when the directions orthogonal to $\boldsymbol{\beta}$ are under stabilizing selection. Specifically, $c(\boldsymbol{\beta})$ quantifies the ability of a multivariate phenotype to change in a $\boldsymbol{\beta}$ direction independently of the variation in the remaining (orthogonal) directions of the phenotypic space, which are assumed to be under stabilizing selection and in equilibrium with $\boldsymbol{\beta}$, so that no response is allowed along any direction other than $\boldsymbol{\beta}$ [8]. The $c(\boldsymbol{\beta})$ is typically lower than $e(\boldsymbol{\beta})$, and these measures will tend to approach

when variation along $\boldsymbol{\beta}$ is genetically uncorrelated with variation along the directions orthogonal to $\boldsymbol{\beta}$ (along which it is assumed that traits will not evolve; [8]).

The evolutionary autonomy in the direction of selection [$a(\boldsymbol{\beta})$] represents the proportion of evolvability along $\boldsymbol{\beta}$ that is left after reduction due to genetic covariances with trait combinations in the remaining directions of the phenotypic space, assumed to be under stabilizing selection. If the variation along $\boldsymbol{\beta}$ is independent of (or not integrated with) variation in other directions, then $a(\boldsymbol{\beta}) = 1$; conversely, if the variation along $\boldsymbol{\beta}$ is completely correlated (or entirely integrated) with variation in other directions, then $a(\boldsymbol{\beta}) = 0$ [8].

Besides $e(\boldsymbol{\beta})$, $c(\boldsymbol{\beta})$ and $a(\boldsymbol{\beta})$, we also assessed the evolutionary flexibility measure proposed by Marroig *et al.* [12], that quantifies the extent to which a **G** matrix deflects the response vector from the direction of the selection gradient vector, and it is estimated by the correlation (i.e. the cosine of the angle) between these vectors. Although not a direct measure of evolvability [9, 13], evolutionary flexibility captures the ability of a population to track with the direction of selection (i.e. a more "flexible" population tracks closer to the direction of selection; [12]).

In a recent study, Hansen *et al.* [9] have indicated that it will be important to estimate both unconditional and conditional evolvabilities when evaluating evolutionary potential, since they may bracket the realized evolvability. However, capturing all relevant constraining variation is unlikely in empirical studies, as it may be impractical to measure a large number of traits. Even under these circumstances, conditional evolvability can still improve our understanding of evolutionary potential by quantifying the constraining effects inherent in the genetic covariance patterns of a given set of measured traits [9]. This can be the case in the present study, as the four measured traits may be able to capture important constraining variation in wood properties, assuming that this variation is indeed structured along a few number of dimensions.

Methods S3: Matrix comparison using the "random skewers" method

To evaluate the difference between the Mainland and Island mean-standardized **G**-matrices in the direction of the response to selection, we quantified the average similarity between the two matrices being compared in their response to a set of random selection gradients (i.e. "random skewers"; [14, 15]). In this approach: (i) each **G** matrix is subjected to the same selection gradient ($\boldsymbol{\beta}$) normalized to unit length; (ii) the vector of trait responses to selection ($\Delta\bar{\mathbf{z}}$) is predicted from the ith **G** matrix as $\Delta\bar{\mathbf{z}}_i = \mathbf{G}_i\boldsymbol{\beta}$; and (iii) the angle (in radians) of response vectors between the two **G** matrices is computed from the inverse cosine of the vector correlation as $\cos^{-1}\left(\frac{\Delta\bar{\mathbf{z}}_1 \bullet \Delta\bar{\mathbf{z}}_2}{\|\Delta\bar{\mathbf{z}}_1\|\|\Delta\bar{\mathbf{z}}_2\|}\right)$, where $\bullet$ and $\| \ \|$ denote the dot product and the vector norm, respectively. After repeating this procedure over a large number of selection gradients of random direction, the mean value of the angles (or, alternatively, the average correlation) of response vectors between the two **G** matrices is calculated, providing a measure of the multivariate similarity in the directions of the response to selection. The mean angle (denoted as $\bar{\varphi}$) will tend to be small when the **G** matrices being compared share a similar pattern of genetic covariance structure, and thus will be indicative of matrix similarity in orientation.

Methods S4: Krzanowski's geometric approach

The Krzanowski geometric approach compares subspaces from different variance-covariance matrices, enabling to evaluate whether the matrices being compared share similar orientation [16, 17, 18]. Following a principal component analysis of each variance-covariance matrix, a subset of k eigenvectors within a q-dimensional trait space (e.g. $q = 4$ in our study) is selected for each matrix to define k-dimensional subspaces for comparison. Ideally, the selected subset of k eigenvectors should explain most of the total variation in the matrices being compared, although k must be $\leq q/2$ [17, 18].

For the comparison of two variance-covariance matrices, let the two k-dimensional subspaces be represented by the matrices **A** and **B** whose columns contain the loadings of the selected eigenvectors. The matrix **S** = **A'BB'A** (with the superscript ' denoting the transpose operation) is then defined for finding the closest alignment between k dimensions of **A** and **B**, with the angles between pairs of best-matching vectors being given by $\cos^{-1}\sqrt{\lambda_n}$, where λ_n is the nth eigenvalue of **S**. These angles measure the extent to which k dimensions of **A** and **B** differ in orientation, and will vary from 0º to 90º (corresponding to λ_i values of 1 and 0, respectively); the minimum angle between two subspaces defined by k dimensions is given by $\cos^{-1}\sqrt{\lambda_1}$, where λ_1 is largest eigenvalue of **S**. In addition, the sum of the eigenvalues of **S** equals the sum of squares of the vector correlations between each of the eigenvectors of **A** and each one of **B**. This sum will vary from 0 to k, providing a bounded index of overall similarity in orientation (denoted as $\Sigma\lambda_S$ in the current study) between the two matrix subspaces; a value of this index approaching 0 indicates that the two subspaces are dissimilar (no shared structure), whereas a value close to k indicates that the two subspaces are coincident (share similar structure) [16, 17, 18].

In the present study, the Krzanowski method was used to pursue a two-dimensional comparison between the Mainland and Island **G**-matrices (see the Materials and Methods), and also between a **G** matrix common to all populations and a phylogenetically-corrected **D** matrix (see Methods S5). The Krzanowski approach is particularly useful to evaluate whether **G** and **D** matrices share a similar orientation as, albeit having equal dimension, the two matrices being compared do not contain the same type of information (i.e. they are not both **G** matrices; e.g. see [19, 20]). Under the applied two-dimensional matrix comparisons, the matrix **S** finds the planes in **A** and **B** that are closest to each other, with the angles $\cos^{-1}\sqrt{\lambda_1}$ and $\cos^{-1}\sqrt{\lambda_2}$ determining the extent to which these planes are non-coincident. In the case of a one-dimensional matrix comparison, $\cos^{-1}\sqrt{\lambda_1}$ is equivalent to the angle between the leading eigenvectors of the matrices

being compared (e.g. the angle between the $\mathbf{g}_{max}$ and $\mathbf{d}_{max}$ axes for a comparison of **G** and **D** matrices).

Methods S5: Comparison of **G** and **D** matrices

To complement the approach based on the estimation of evolvability along the direction of population divergence, we also evaluated the putative influence of genetic architecture on population differentiation by comparing the pooled within-population **G-**matrix with a variance-covariance matrix summarizing the divergence in multivariate phenotype among populations (**D**) (e.g. [20, 21]). Instead of obtaining the latter matrix from trait (co)variances based on phenotypic means estimated for populations, we modified the model described in Equation (1) (see the Materials and methods) to fit populations as a random term (rather than a fixed effect), and thus to construct **D** from REML estimates of trait (co)variances. This has the advantage of controlling for environmental effects (captured by experimental design features - replicates and incomplete blocks - and also by the residual term in the model) in estimating population (co)variances, while also enabling access to their sampling error via the inverse of the Average Information matrix.

Estimation of **D** adjusted for phylogenetic relationships among populations will be preferable when populations share a common history [22, 23]. To account for non-independence of populations due to common ancestry, we incorporated a phylogenetic relatedness matrix for modelling population (phylogenetic) effects fitted as a random term (hereafter denoted by the vector $\mathbf{u}_p$) in the linear mixed model [4, 24, 25] . In this context, the distribution of the effects in $\mathbf{u}_p$ was assumed to be multivariate normal with a zero-mean vector and a variance matrix defined as $\mathbf{D} \otimes \mathbf{C}$, where $\mathbf{D}$ is a matrix of phylogenetic-corrected among-population (co)variances and $\mathbf{C}$ is a phylogenetic relatedness matrix. The (co)variances in $\mathbf{D}$ were estimated by excluding a random term for a residual population effect in the mixed model (e.g. [4]); yet, previous univariate analyses of each trait indicated that the variance associated with this term was

either zero or not statistically significant according to a likelihood-ratio (LR) test (not shown). Based on an ultrametric tree obtained from the reconstructed phylogeny of the populations (Methods S1; Figure S1), the **C** matrix was estimated by using the *vcvPhylo* function in the R-package Phytools [7], assuming Brownian motion. **C** was incorporated in ASReml as a correlation matrix (so that its diagonal elements pertain to the root-to-tip length in the phylogeny scaled to unity, and the off-diagonals refer to the shared branch lengths between the populations), and then inverted by ASReml to model the effects in $\mathbf{u}_p$. Overall trait means estimated from this mixed model correspond to the inferred ancestral states for the traits (which were alternatively estimated by using the *fastAnc* function in Phytools). LR tests were applied to assess the statistical significance of the estimated population (co)variances and correlations.

The pooled **G-**matrix estimated from the initial mixed model remained virtually unchanged when populations were modelled as random effects (as described above), and thus it was used for comparison with the phylogenetically-adjusted **D** matrix to be consistent with previous analyses. A geometric approach was undertaken to compare these matrices in terms of their size, shape and orientation. Following principal component analysis of each matrix, the sum of all eigenvalues (i.e. the total variance, which equals the trace of a matrix when it is positive definite) of a matrix provided a descriptor of its size; shape was described by a measure of matrix eccentricity, calculated as the ratio of the first eigenvalue to the total variance (e.g. Equations (2) and (3) in [26]). The Krzanowski geometric approach [16, 17, 18] was applied to evaluate whether the two matrices had similar orientation (see the Materials and Methods, and Methods S4, for further details on this method). Under this approach, the first two eigenvectors of the **G** and **D** matrices (accounting, respectively, for 95% and 99% of the total variance in each matrix; Table S8) defined the subspaces to be used in a two-dimensional matrix comparison (k = 2). We also pursued a one-dimensional comparison between the two matrices, which is equivalent to calculating the angle between their leading eigenvectors ($\mathbf{g}_{\max}$ and $\mathbf{d}_{\max}$), hence evaluating whether populations have diverged close to the direction of a genetic line of least resistance. Both

G and **D** matrix estimates were compared on a common scale, via their mean-standardization using the estimated phylogenetically-weighted trait means (i.e. inferred ancestral states).

Methods S6: REML-MVN sampling approach

The REML-MVN sampling approach assumes that the REML (co)variance estimates from a linear mixed model asymptotically have a multivariate normal distribution, with means given by the vector of REML parameter estimates at convergence ($\hat{\boldsymbol{\theta}}$) and a (co)variance matrix approximated by the inverse of the Average Information matrix ($\mathbf{H}(\hat{\boldsymbol{\theta}})^{-1}$) [27, 28] (see also [29, 30, 31]); both $\hat{\boldsymbol{\theta}}$ and $\mathbf{H}(\hat{\boldsymbol{\theta}})^{-1}$ are provided by ASReml. Thus, samples of a variance-covariance matrix that had been estimated by REML can be obtained by directly sampling its elements from the distribution $N(\hat{\boldsymbol{\theta}},\ \mathbf{H}(\hat{\boldsymbol{\theta}})^{-1})$. However, such direct sampling procedure may result in matrix samples with (co)variance values outside of the theoretical parameter space, particularly when an estimated variance-covariance matrix has eigenvalues approaching zero.

Alternatively to a direct sampling procedure, REML-MVN sampling can be performed on the elements of the lower triangular matrix **L** pertaining to the Cholesky factors of an estimated variance-covariance matrix, and those sampled elements of **L** are then used to construct samples (obtained from $\mathbf{LL}'$) of the estimated matrix that are positive semi-definite [27, 28]. Analogously to this sampling on the **L**-scale, we pursued REML-MVN sampling on the elements of the factors obtained from the parameterization of a (unstructured) variance-covariance matrix as a reduced-rank factor analytic (FA) structure. In this context, for the effects in $\mathbf{u}_f$, the standard multiple-trait variance-covariance matrix (described in Equation (2); see the Materials and methods) was parameterized by an equivalent model that used a FA structure of order 4 (equal to the number of traits) with the trait specific variances set to zero [32, 33]. This was also done for population effects in the case where the model described in Equation (1) was modified to fit populations as a random term (i.e. estimation of the among-population **D** matrix; Methods S5). ASReml was used

to model the FA structure, and to provide the inverse of the Average Information matrix from where the sampling (co)variances of the relevant parameter estimates (i.e. factor loadings) were obtained. We then applied the REML-MVN sampling approach to construct 100,000 samples of either **G** or **D** matrix estimates, calculated the target measures for each sample, and generated the sampling distribution for each measure across samples.

Constraints on the parameter space associated with sampling on the **L**-scale ensures that matrix samples are positive semi-definite, but (in contrast to direct sampling on matrix elements) may lead to means of the sampling distributions of the (co)variance parameters that differ from their REML estimates [27, 28]. In this context, the relative bias we have observed (using sampling distributions based on 100,000 REML-MVN matrix samples) was fairly small for our estimated variance-covariance matrices (i.e. in general, less than 10% for individual parameter estimates, and with a bias averaged over parameters not exceeding 5% for any of the **G** matrices estimated within or across the Mainland and Island groups, as well as for the estimated among-population **D** matrix). In addition, both the boundary constraints and the concentration of sampling variance in leading eigenvalues may result in their overestimation [31]. Nevertheless, in general, the means we have observed in our generated sampling distributions of eigenvalues (again based on 100,000 REML-MVN matrix samples) did not differ substantially in relation to the corresponding values obtained from the principal component analysis of the estimated variance-covariance matrices (e.g. relative differences of 9%, 7% and 4% for the leading eigenvalue of the **G** matrices estimated for the Mainland, Island and all populations across the two groups, respectively, and 8% for the leading eigenvalue of the estimated among-population **D** matrix).

Methods S7: Statistical support for similarity of two estimated (co)variance matrices

Evaluating the statistical support for similarity of two estimated variance-covariance matrices being compared depended on whether measures were computed separately for each matrix and then compared between matrices (i.e. measures that capture the potential for evolution; measures of matrix size and shape), or were directly calculated from a between-matrix comparison (i.e. $\bar{\varphi}$, $\Sigma\lambda_S$ and angle between $\mathbf{g}_{max}$ and $\mathbf{d}_{max}$) [34].

For a measure computed separately for each matrix and then compared between matrices, we generated the sampling distribution for the difference between matrices, based on 100,000 REML-MVN matrix samples of either estimated matrix. In particular, for a multivariate measure of evolutionary potential compared between the Mainland and Island groups, the generated sampling distribution referred to a difference in means (i.e. $\bar{e}$, $\bar{c}$, $\bar{a}$, or $\bar{f}$), with each mean being computed over 5000 unit-length random selection gradients (as described in the Materials and Methods) for a given REML-MVN matrix sample drawn from the estimated **G** matrix of either group. Based on a sampling distribution generated from 100,000 observations, the 95% confidence interval for the difference between matrices in a measure was then approximated: when overlapping with zero, this 95% confidence interval will be indicative of statistical support for similarity between the estimated matrices in the measure under consideration.

For a measure *m* directly computed from a between-matrix comparison, the statistical support for similarity of two estimated (co)variance matrices - denoted here as $\mathbf{C}_1$ and $\mathbf{C}_2$ for descriptive purposes - can be assessed by comparing estimates of *m* obtained within $\mathbf{C}_1$ and $\mathbf{C}_2$ (in which case any difference of *m* from complete matrix similarity is expected to reflect sampling error only) with *m* estimated between $\mathbf{C}_1$ and $\mathbf{C}_2$ [34, 35]. In this sense, pairs of samples (*a* and *b*) from both $\mathbf{C}_1$ and $\mathbf{C}_2$ are used to calculate the following statistic:

$$\Psi_m(\mathbf{C}_1, \mathbf{C}_2) = [\, m(\mathbf{C}_1^a, \mathbf{C}_1^b) + m(\mathbf{C}_2^a, \mathbf{C}_2^b)\,] - [\, m(\mathbf{C}_1^a, \mathbf{C}_2^a) + m(\mathbf{C}_1^b, \mathbf{C}_2^b)\,]$$

where the first term of the right-hand side of the equation refers to the measure *m* estimated from samples of the same matrix, and the second term pertains to the measure *m* estimated from samples of different matrices [35, 36]. Thus, for a given measure *m* (i.e. $\overline{\varphi}$, $\Sigma\lambda_S$ or angle between $\mathbf{g}_{max}$ and $\mathbf{d}_{max}$), $\Psi_m(\mathbf{C}_1, \mathbf{C}_2)$ evaluates whether differences within $\mathbf{C}_1$ and $\mathbf{C}_2$ due to sampling error are similar to differences between $\mathbf{C}_1$ and $\mathbf{C}_2$. For either estimated matrix being compared, 100,000 pairs of REML-MVN matrix samples were drawn, and Ψ_m was then calculated for each generated pair. Based on a sampling distribution generated from 100,000 observations, the 95% confidence interval for Ψ_m was then approximated: when overlapping with zero, this 95% confidence interval will be indicative of statistical support for similarity between the estimated matrices in the measure *m* under consideration.

2. Supplementary results

Results S1: Comparison of **G** and **D** matrices

Table S5 presents the phylogenetically-corrected **D** matrix, describing the actual among-population (co)evolution that has occurred for the traits, together with the **G** matrix common to all populations. The phylogenetically-corrected variances in **D** represent evolutionary rates for the traits (i.e. their mean-scaled variances accumulated over the length of the phylogeny; e.g. [4, 23]), with the descriptor of matrix size (i.e. the trace of **D**) providing a measure of the overall rate of population diversification in the four-trait phenotypic space. Highly significant ($P < 0.001$) variances were detected for all traits in both **G** and **D** (Table S5). Statistical support for similarity in matrix size could not be rejected (Figure S3a), although the total variance in **D** was slightly higher than that in **G** (Table S9). Thus, in general, the mean-scaled variances in **D** were not large when compared to the corresponding estimates in **G**. However, this pattern was not apparent for the trait covariances, which tended to be stronger in **D** (Table S5). This was reflected in the descriptor of matrix shape, which provided 94% and 84% for the percentage of the total variance being accounted for by the first eigenvector of **D** and **G**, respectively (Tables S8 and S9). Similarity in matrix shape was not statistically supported, as the 95% CI for the difference between matrices in this measure did not include zero (Figure S3b), and thus indicated that **G** and **D** were not proportional. This suggests that genetic drift may not have played an important role in generating population differentiation, as **G** and **D** are expected to be proportional under neutral divergence (see Discussion). The high eccentricity of the **D** matrix, indicated by the large concentration of variance in the main axis of among-population variation, suggests a narrow range of directions along which populations have diverged. Such pattern of population diversification may reflect the uneven distribution of the genetic variation in **G** (Table S8), which may have limited the amount of highly evolvable directions in the phenotypic space, as indicated

by $\bar{a} \approx 0.3$ (Table S6). In addition, the moderate level of average flexibility (i.e. $\bar{f} \approx 0.6$; Table S6) associated with **G** indicated a reduced ability for response to a broad range of directions of selection.

Although having a stronger magnitude in **D**, the covariance estimates showed that the traits co-varied in a similar direction in the **G** and **D** matrices (Table S5). This considerable resemblance in trait relationships was reflected in the high level of similarity between **G** and **D** in orientation. Under a two-dimensional matrix comparison using the Krzanowski method, the index $\Sigma\lambda_S$ of overall similarity was 1.91 (95% CI: 1.66, 1.98) of a possible 2 (Table S9). The $\mathbf{g}_{max}$ and $\mathbf{d}_{max}$ directions were closely aligned, with an angle of 5.9° (95% CI: 2.6°, 11.8°) between them. Statistical support for similarity in matrix orientation could not be rejected for either of these two measures (Figures S3c and S3d). In addition, the angle between the second eigenvectors of **G** and **D** was also relatively small (i.e. 17.5°; 95% CI: 9.3°, 35.9°), with the 95% CI of the statistic Ψ_m including zero (not shown), and thus providing statistical support for shared orientation between these two axes. These results suggest that axes of greatest within-population genetic (co)variance may have determined trajectories of evolutionary change, with population divergence occurring mainly close to the direction of a genetic line of least resistance.

3. Supplementary figures

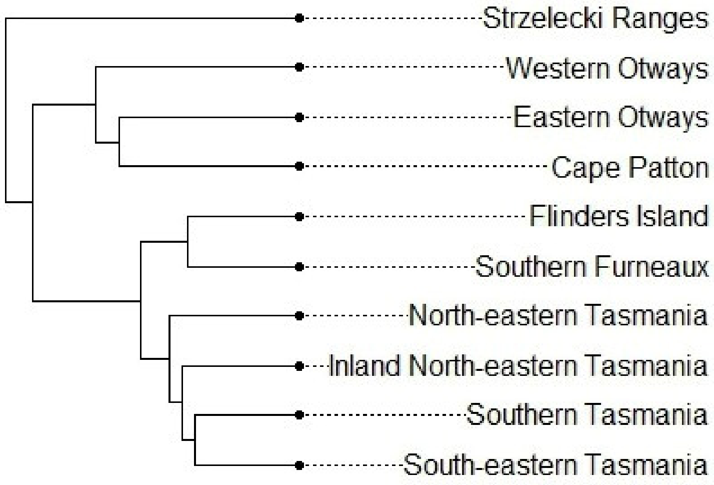

Figure S1. The phylogenetic tree generated for the ten studied populations of *Eucalyptus globulus* using 812,158 genome-wide SNPs. The tree was derived from Nei's genetic distance matrix by using the neighbour-joining method, as detailed in Methods S1. See Table S1 for the identification of the studied populations within the Mainland and Island groups, as well as the number of families and trees measured per population.

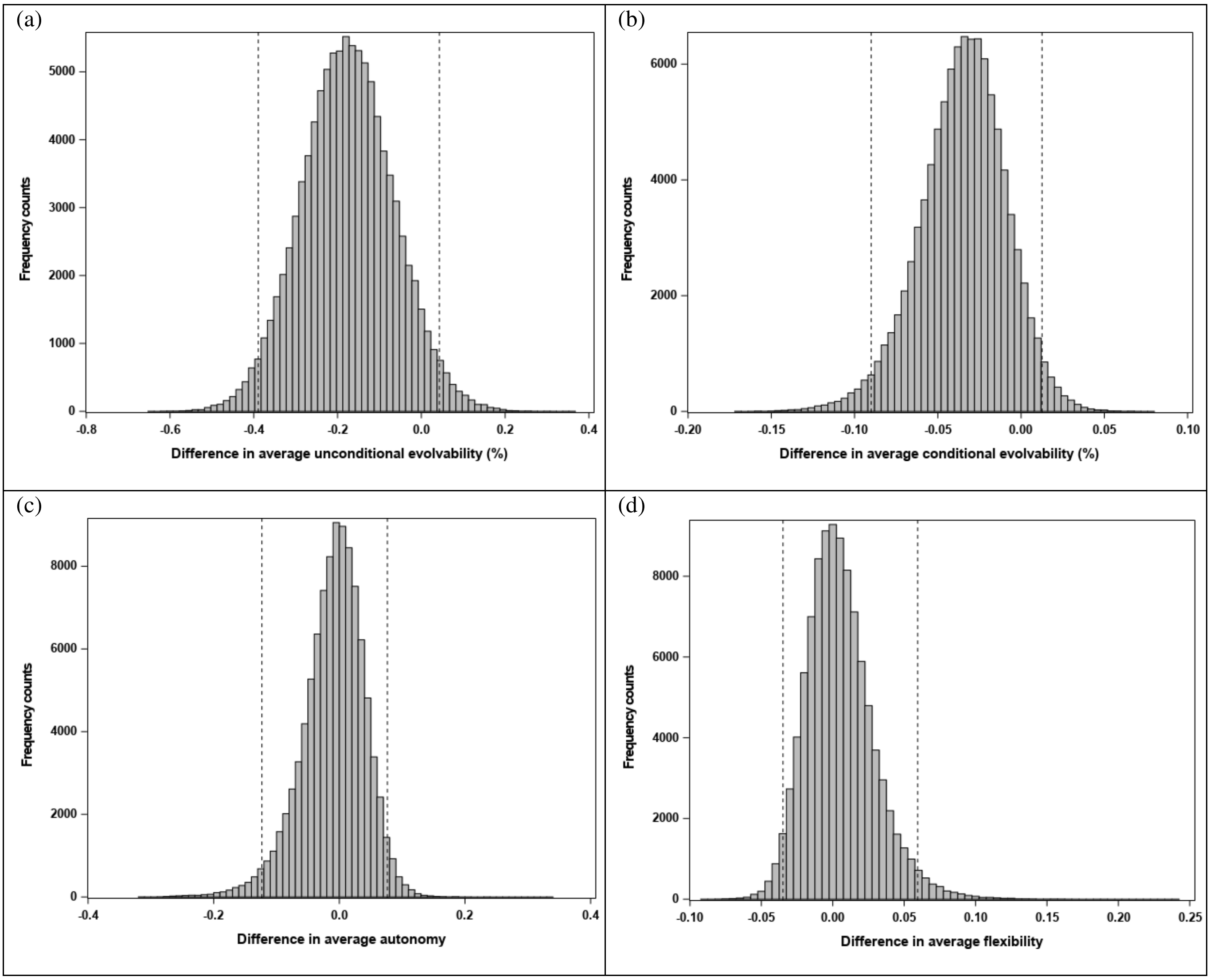

Figure S2. Simulated sampling distributions and 95% confidence intervals (depicted by the dashed vertical lines), obtained by the REML-MVN sampling approach [27, 28], for the difference between the Mainland and Island population groups in mean values of the following multivariate measures that capture the potential for evolution: (a) unconditional evolvability; (b) conditional evolvability; (c) autonomy; and (d) flexibility. These measures were calculated separately for each group, based on the corresponding mean-standardized **G**-matrix (see Table 1, and the Materials and Methods, for further details). When overlapping with zero, a 95% confidence interval for the difference between groups in a given measure indicates statistical support for similarity between the two matrices being compared.

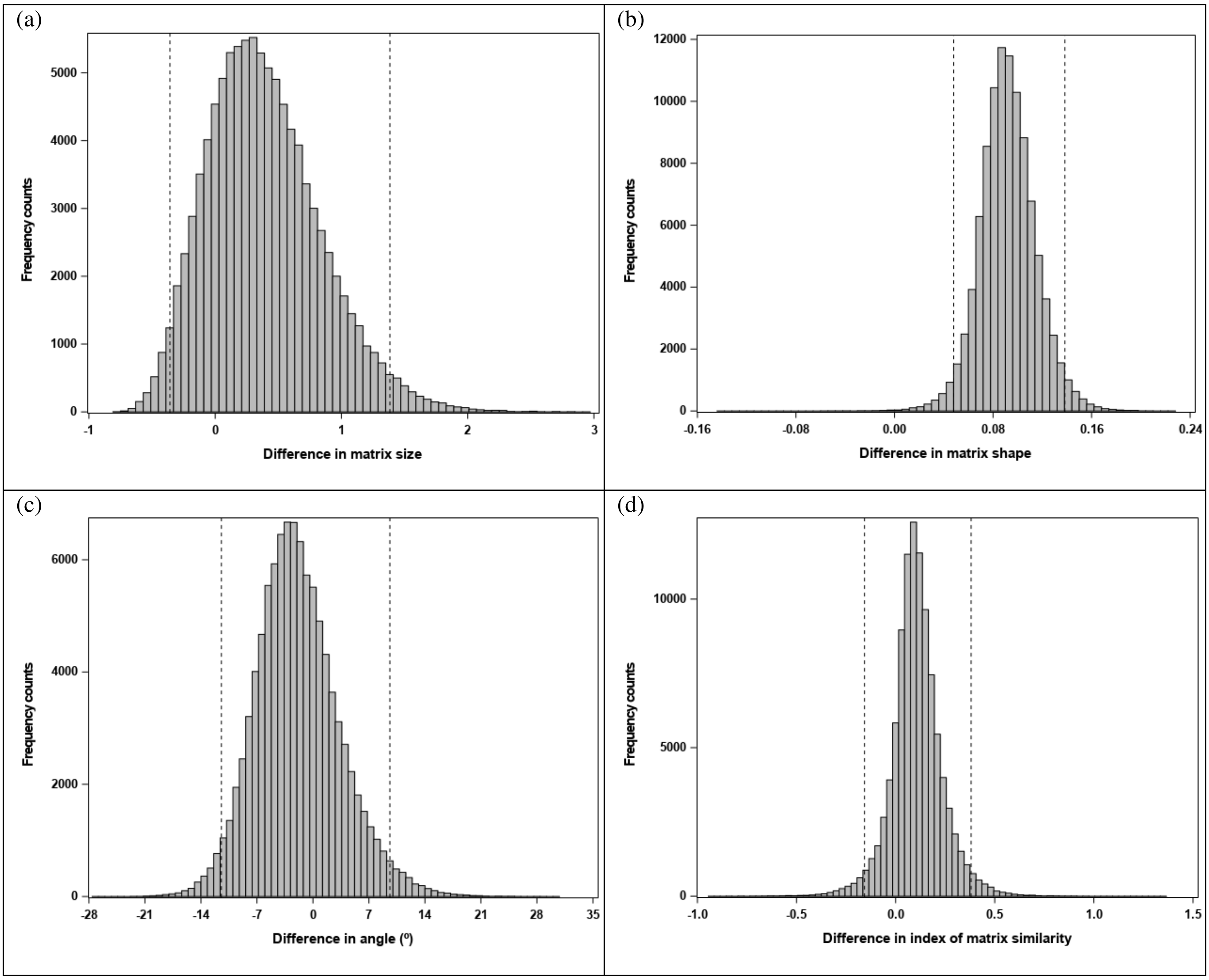

Figure S3. Simulated sampling distributions and 95% confidence intervals (depicted by the dashed vertical lines), obtained by the REML-MVN sampling approach [27, 28], for the differences in measures of matrix size and shape, and for the statistic Ψ_m evaluating similarity in matrix orientation, used to compare the mean-standardized additive genetic (**G**) and among-population (**D**) variance-covariance matrices estimated across all studied populations. Plots (a) and (b) refer to the difference between **G** and **D** in descriptors of matrix size and shape, respectively, calculated separately for each matrix. Plots (c) and (d) pertain to the statistic Ψ_m used to assess whether the **G** and **D** matrices had a similar orientation based, respectively, on the following measures directly computed from a between-matrix comparison: angle between $\mathbf{g}_{max}$ and $\mathbf{d}_{max}$; and Krzanowski's index of overall similarity in orientation between matrix subspaces. For each of these two latter measures, the statistic Ψ_m evaluates whether differences within matrices due to sampling error are similar to differences between matrices (see Methods S7). A given 95% confidence interval that overlaps with zero provides indication of similarity between the two matrices being compared for the aspect (size, shape or orientation) of interest.

4. Supplementary tables

Table S1. Mainland and Island population groups of *E. globulus*: identification of the studied populations within groups, and number of families and trees measured per population.

Population group	Population	Number of families	Number of measured trees
Mainland	Cape Patton	16	72
	Eastern Otways	23	112
	Strzelecki Ranges	57	263
	Western Otways	107	482
Island	Flinders Island	48	216
	Southern Furneaux	45	204
	Inland North-eastern Tasmania	18	86
	North-eastern Tasmania	16	73
	South-eastern Tasmania	53	238
	Southern Tasmania	25	111

Table S2. Means, phenotypic standard deviations ($\sqrt{\hat{\sigma}_p^2}$), mean-scaled within-family variance (I_W) and narrow-sense heritabilities ($\hat{h}^2$) for wood property traits (S/G, KL, BD and EX) measured in *E. globulus*, and estimated for the Mainland and Island population groups.

	Mainland group				Island group			
	Mean	$\sqrt{\hat{\sigma}_p^2}$	I_W	$\hat{h}^2$	Mean	$\sqrt{\hat{\sigma}_p^2}$	I_W	$\hat{h}^2$
S/G	1.9	0.124	0.27 ± 0.03	0.37 (± 0.08)	2.0	0.114	0.18 ± 0.03	0.42 (± 0.08)
KL (%)	20.8	0.955	0.16 ± 0.02	0.26 (± 0.08)	20.2	0.929	0.15 ± 0.02	0.28 (± 0.08)
BD (kg/m^3)	549.4	33.53	0.22 ± 0.03	0.42 (± 0.08)	532.8	31.89	0.18 ± 0.03	0.49 (± 0.09)
EX (%)	5.3	1.29	4.5 ± 0.5	0.22 (± 0.08)	4.3	0.95	2.8 ± 0.4	0.41 (± 0.08)

Traits: S/G = syringyl to guaiacyl ratio; KL = lignin (Klason) content; BD = basic density; EX = extractive content. All the estimates presented in the table pertain to unstandardized data, except I_W (with values multiplied by 100) which refers to mean-standardized data via the trait means within a population group. The I_W were estimated from $\hat{\sigma}_e^2 - 0.6 I_A$ (see alternative formula in Fig. 8 in [10]), where $\hat{\sigma}_e^2$ is the residual variance estimated from the model described in Equation (1) and I_A is the univariate mean-scaled evolvability (estimated by using a coefficient of relationship between OP-sibs of 0.4; see the Materials and Methods); I_W will approximate the mean-scaled variance associated with micro-site environmental effects on individual trees, under the assumption that environmental variance is strong relative to the variance related to non-additive genetic effects (i.e. dominance and epistasis). The standard errors of $\hat{h}^2$ (within parentheses) were approximated by using the Delta method.

Table S3. Principal component (PC) analysis of the additive genetic (**G**) variance-covariance matrices estimated for wood traits (S/G, KL, BD and EX) within the Mainland and Island population groups of *E. globulus*. The variance accounted for by each PC is indicated by the eigenvalue (with the value within parentheses referring to the percentage of the total variance explained by the PC), and the eigenvector shows the component loadings on each of the wood property traits.

	Mainland **G**-matrix				Island **G**-matrix			
	PC1	PC2	PC3	PC4	PC1	PC2	PC3	PC4
Eigenvalue [a]	1.410 (86.3 %)	0.154 (9.4 %)	0.050 (3.1 %)	0.020 (1.2 %)	2.017 (86.3 %)	0.224 (9.6 %)	0.069 (2.9 %)	0.028 (1.2 %)
Eigenvector								
S/G	-0.282	-0.110	0.950	0.073	-0.104	-0.527	0.814	-0.220
KL	0.158	-0.075	-0.037	0.984	0.104	-0.210	0.137	0.962
BD	0.065	0.987	0.128	0.070	-0.039	0.823	0.557	0.105
EX	0.944	-0.088	0.282	-0.148	0.988	-0.001	0.093	-0.121

Traits: S/G = syringyl to guaiacyl ratio; KL = lignin (Klason) content; BD = basic density; EX = extractive content. For each population group, **G** was estimated as a single matrix to represent the populations within the group. The results are based on mean-standardized **G** matrices via the trait means within a population group (see the Material and Methods).

[a] The eigenvalues are multiplied by 100.

Table S4. Pearson correlations (with significance probabilities in parentheses) between the estimated **G**-matrices of the Mainland and Island groups for multivariate measures reflecting evolutionary potential along different random directions.

e	*c*	*a*	*f*
0.91 ($P < 0.001$)	0.89 ($P < 0.001$)	0.83 ($P < 0.001$)	0.72 ($P < 0.001$)

Unconditional evolvability (*e*), conditional evolvability (*c*), autonomy (*a*) and flexibility (*f*) were computed along 5000 unit-length random selection gradients uniformly distributed in a 4-dimensional space. For each group, **G** was estimated as a single, mean-standardized matrix to represent the populations within the group. Prior to assessing the Pearson correlation, the 5000 observations for each measure were transformed to a normal shape by using a rank-based inverse normal transformation (i.e. transformation to rankit scores), as suggested by Bishara and Hittner [37].

Table S5. Additive genetic (**G**) and population divergence (**D**) variance-covariance matrices estimated for wood traits (S/G, KL, BD and EX) across all the studied populations of *E. globulus*. Phylogenetically-corrected (co)variances are provided in **D**. Parameter estimates are given together with their standard errors for variances (diagonal), covariances (below diagonal) and correlations (above diagonal).

	S/G	KL	BD	EX
***G**-matrix common to all populations*				
S/G	0.142 ± 0.024 ($P < 0.001$)	-0.28 ± 0.12 ($P = 0.033$)	-0.31 ± 0.10 ($P = 0.005$)	-0.57 ± 0.10 ($P < 0.001$)
KL	-0.026 ± 0.012 ($P = 0.033$)	0.059 ± 0.012 ($P < 0.001$)	-0.18 ± 0.12 ($P > 0.05$)	0.70 ± 0.08 ($P < 0.001$)
BD	-0.048 ± 0.017 ($P = 0.005$)	-0.018 ± 0.012 ($P > 0.05$)	0.166 ± 0.024 ($P < 0.001$)	0.01 ± 0.12 ($P > 0.05$)
EX	-0.256 ± 0.064 ($P < 0.001$)	0.203 ± 0.050 ($P < 0.001$)	0.005 ± 0.060 ($P > 0.05$)	1.443 ± 0.294 ($P < 0.001$)
***D**-matrix of population divergence*				
S/G	0.145 ± 0.072 ($P < 0.001$)	-0.53 ± 0.26 ($P > 0.05$) [a)]	-0.80 ± 0.14 ($P = 0.006$)	-0.81 ± 0.13 ($P = 0.004$)
KL	-0.055 ± 0.041 ($P > 0.05$)	0.074 ± 0.037 ($P < 0.001$)	0.03 ± 0.36 ($P > 0.05$)	0.86 ± 0.10 ($P = 0.001$)
BD	-0.088 ± 0.049 ($P = 0.006$)	0.002 ± 0.028 ($P > 0.05$)	0.083 ± 0.043 ($P < 0.001$)	0.43 ± 0.30 ($P > 0.05$)
EX	-0.415 ± 0.229 ($P = 0.004$)	0.314 ± 0.169 ($P = 0.001$)	0.168 ± 0.150 ($P > 0.05$)	1.796 ± 0.898 ($P < 0.001$)

Traits: S/G = syringyl to guaiacyl ratio; KL = lignin (Klason) content; BD = basic density; EX = extractive content. All the (co)variance estimates presented in the table are multiplied by 100, and pertain to mean-standardized matrices via the phylogenetically-weighted trait means (see Methods S5). Significance probabilities from likelihood ratio tests are given within parenthesis (note that testing a variance estimate was pursued by fitting a univariate model that ignored the trait covariances, in order to avoid estimation and convergence problems that could arise with a multivariate model when constraining a variance estimate to remain fixed at zero under the null hypothesis).

[a)] $P \approx 0.10$.

Table S6. Mean values of unconditional evolvability ($\bar{e}$), conditional evolvability ($\bar{c}$), autonomy ($\bar{a}$) and flexibility ($\bar{f}$) (with 95 % confidence intervals within parentheses) based on the estimated **G**-matrix common to all populations of *E. globulus*.

$\bar{e}$ [a)]	$\bar{c}$ [a)]	$\bar{a}$	$\bar{f}$
0.452<br>(0.340, 0.611)	0.111<br>(0.084, 0.135)	0.326<br>(0.242, 0.395)	0.627<br>(0.582, 0.676)

The $\bar{e}$, $\bar{c}$, $\bar{a}$ and $\bar{f}$ mean values were computed over 5000 unit-length random selection gradients uniformly distributed in a 4-dimensional space. The estimated **G**-matrix common to all populations (i.e. a pooled matrix obtained by combining data across all the open-pollinated families nested within the studied populations) was mean-standardized via the phylogenetically-weighted trait means (see the Materials and Methods, and Methods S5).

[a)] The $\bar{e}$ and $\bar{c}$ values are multiplied by 100.

Table S7. Amount of divergence from the inferred ancestral states, unconditional ($e(\mathbf{z})$) and conditional ($c(\mathbf{z})$) evolvabilities along the direction of divergence ($\mathbf{z}$), and angle between $\mathbf{z}$ and $\mathbf{g}_{max}$, for the studied populations of *E. globulus*.

Population	Amount of divergence (%)	$e(\mathbf{z})$ (%)	$c(\mathbf{z})$ (%)	Angle between $\mathbf{z}$ and $\mathbf{g}_{max}$ (°) [a)]
Western Otways	1.46	0.290	0.086	69.4
Cape Patton	5.02	1.410	0.603	16.5
North-eastern Tasmania	7.27	1.264	0.462	25.7
Strzelecki Ranges	11.65	1.394	0.589	17.6
Eastern Otways	11.66	1.416	0.882	16.1
Flinders Island	14.27	1.516	1.417	3.2
Inland North-eastern Tasmania	17.08	1.203	0.492	29.1
South-eastern Tasmania	17.79	1.359	0.708	20.3
Southern Furneaux	18.17	1.514	1.359	3.8
Southern Tasmania	27.54	1.500	1.131	7.0

For details on the estimation of the amount of divergence from the inferred ancestral states, and $e(\mathbf{z})$ and $c(\mathbf{z})$ along the $\mathbf{z}$-direction, see Table 2 and the Materials and Methods. The $\mathbf{g}_{max}$ direction corresponds to the first eigenvector of the **G**-matrix common to all populations (Table S8). The angle between $\mathbf{z}$ and $\mathbf{g}_{max}$ was calculated by $[\cos^{-1}(\mathbf{z}, \mathbf{g}_{max})]180/\pi$, where the two vectors are normalized to unit length, and ' denotes the transpose operator.

[a)] The actual angle between a divergence vector ($\mathbf{z}$) and $\mathbf{g}_{max}$ may vary from 0° (completely aligned) to 90° (orthogonal). To evaluate whether the directions of population divergence were significantly aligned with $\mathbf{g}_{max}$, we compared each of these angles to a null distribution that was generated by simulating 100,000 pairs of random vectors uniformly distributed in a 4-dimensional space (i.e. equal to the number of measured traits). The elements of each of these vectors were randomly drawn from a normal distribution with a mean of 0 and a variance of 1, and then each vector was normalized to unit length. The critical values of the null distribution were 28.6°, 16.6° and 8.2°, corresponding to the 5th, 1th and 0.1th percentiles of the simulated distribution, respectively. Thus, all populations except Western Otways appeared to be well aligned with $\mathbf{g}_{max}$ (although for Inland North East Tasmania the alignment was marginally non-significant, i.e. 29.1° versus 28.6°).

Table S8. Principal component (PC) analysis of the additive genetic (**G**) and population divergence (**D**) variance-covariance matrices estimated for wood traits (S/G, KL, BD and EX) across all the studied populations of *E. globulus*. The variance accounted for by each PC is indicated by the eigenvalue (with the value within parentheses referring to the percentage of the total variance explained by the PC), and the eigenvector shows the component loadings on each of the wood property traits.

	**G**-matrix common to all populations				**D**-matrix of population divergence			
	PC1	PC2	PC3	PC4	PC1	PC2	PC3	PC4
Eigenvalue [a)]	1.520<br>(84.0 %)	0.191<br>(10.6 %)	0.071<br>(3.9 %)	0.027<br>(1.5 %)	1.966<br>(93.7 %)	0.116<br>(5.5 %)	0.012<br>(0.6 %)	0.005<br>(0.2 %)
Eigenvector								
S/G	-0.183	-0.429	0.880	-0.089	-0.227	-0.571	0.659	0.434
KL	0.138	-0.140	0.060	0.979	0.165	-0.341	-0.664	0.645
BD	0.008	0.890	0.446	0.099	0.096	0.731	0.255	0.625
EX	0.973	-0.069	0.153	-0.157	0.955	-0.150	0.246	-0.071

Traits: S/G = syringyl to guaiacyl ratio; KL = lignin (Klason) content; BD = basic density; EX = extractive content. **G** was estimated as a additive genetic variance-covariance matrix common to all populations (i.e. a pooled matrix obtained by combining data across all the open-pollinated families nested within the studied populations). **D** refers to a matrix of population divergence with phylogenetically-corrected (co)variances. Both matrices were mean-standardized via the phylogenetically-weighted trait means (see Methods S5).

[a)] The eigenvalues are multiplied by 100.

Table S9. Comparison of the additive genetic (**G**) and population divergence (**D**) variance-covariance matrices estimated across all studied populations of *E. globulus*, based on descriptors of matrix size and shape, and on measures of matrix similarity in orientation (with 95% confidence intervals within parentheses). Phylogenetically-corrected (co)variances were estimated in **D**.

	Size [a)]	Shape	Orientation	
			Angle between $\mathbf{g}_{max}$ and $\mathbf{d}_{max}$ (°)	Krzanowski's index ($\Sigma\lambda_S$) of overall similarity
**G**-matrix common to all populations	1.8 (1.3, 2.4)	0.84 (0.78, 0.89)	5.9 (2.6, 11.8)	1.91 (1.66, 1.98)
**D**-matrix of population divergence	2.1 (1.1, 3.6)	0.94 (0.86, 0.97)		

Descriptors of matrix size and shape were calculated separately for each matrix, whereas similarity in matrix orientation was quantified by measures directly computed from a between-matrix comparison. Both matrices were mean-standardized via the phylogenetically-weighted trait means (see Methods S5). See Figure S3 to evaluate the statistical support for similarity of the **G** and **D** matrices in the measures provided in the table.

[a)] The eigenvalues were multiplied by 100 in the calculation of the descriptor of matrix size.

5. Supplementary references

1. Thavamanikumar, S.; McManus, L.J.; Ades, P.K.; Bossinger, G.; Stackpole, D.J.; Kerr, R.; Hadjigol, S.; Freeman, J.S.; Vaillancourt, R.E.; Zhu, P.; Tibbits, J.F.G. Association mapping for wood quality and growth traits in *Eucalyptus globulus* ssp. *globulus* Labill identifies nine stable marker-trait associations for seven traits. *Tree Genetics and Genomes* **2014**, *10*, 1661-1678.
2. Nei, M. Genetic distance between populations. *The American Naturalist* **1972**, *106*, 283-292.
3. Pembleton, L.W.; Cogan, N.O.I.; Forster, J.W. StAMPP: an R package for calculation of genetic differentiation and structure of mixed-ploidy level populations. *Molecular Ecology Resources* **2013**, *13*, 946-952.
4. Bolstad, G.H.; Hansen, T.F.; Pélabon, C.; Falahati-Anbaran, M.; Pérez-Barrales, R.; Armbruster, W.S. Genetic constraints predict evolutionary divergence in Dalechampia blossoms. *Philosophical Transactions of the Royal Society B: Biological Sciences* **2014**, *369*, 20130255.
5. Jones, R.C.; Steane, D.A.; Lavery, M.; Vaillancourt, R.E.; Potts, B.M. Multiple evolutionary processes drive the patterns of genetic differentiation in a forest tree species complex. *Ecology and Evolution* **2013**, *3*, 1-17.
6. Jones, R.C.; Nicolle, D.; Steane, D.A.; Vaillancourt, R.E.; Potts, B.M. High density, genome-wide markers and intra-specific replication yield an unprecedented phylogenetic reconstruction of a globally significant, speciose lineage of Eucalyptus. *Molecular Phylogenetics and Evolution* **2016**, *105*, 63-85.
7. Revell, L.J. phytools: an R package for phylogenetic comparative biology (and other things). *Methods in Ecology and Evolution* **2012**, *3*, 217-223.
8. Hansen, T.F.; Houle, D. Measuring and comparing evolvability and constraint in multivariate characters. *Journal of Evolutionary Biology* **2008**, *21*, 1201-1219.
9. Hansen, T.F.; Solvin, T.M.; Pavlicev, M. Predicting evolutionary potential: A numerical test of evolutionary measures **2019**, Evolution, *73*, 689-703.
10. Hansen, T.F.; Pélabon, C.; Houle, D. Heritability is not evolvability. *Evolutionary Biology* **2011**, *38*, 258.

11. Hansen, T.F.; Armbruster, W.S.; Carlson, M.L.; Pélabon, C. Evolvability and genetic constraint in *Dalechampia* blossoms: genetic correlations and conditional evolvability. *Journal of Experimental Zoology* **2003**, *296*, 23-39.
12. Marroig, G.; Shirai, L.T.; Porto, A.; de Oliveira, F.B.; De Conto, V. The evolution of modularity in the mammalian skull II: Evolutionary consequences. *Evolutionary Biology* **2009**, *36*, 136-148.
13. Armbruster, W.S.; Pélabon, C.; Bolstad, G.H.; Hansen, T.F. Integrated phenotypes: understanding trait covariation in plants and animals. *Philosophical Transactions of the Royal Society B: Biological Sciences* **2014**, *369*, 20130245.
14. Cheverud, J.M. Quantitative genetic analysis of cranial morphology in the cotton-top (*Saguinus oedipus*) and saddle-back (*S. fuscicollis*) tamarins. *Journal of Evolutionary Biology* **1996**, *9*, 5-42.
15. Cheverud, J.M.; Marroig, G. Comparing covariance matrices: random skewers method compared to the common principal components model. *Genetics and Molecular Biology* **2007**, *30*, 461-469.
16. Krzanowski, W.J. Between-groups comparison of principal components. *Journal of the American Statistical Association* **1979**, *74*, 703-707.
17. Krzanowski, W.J. *Principles of Multivariate Analysis: A User's Perspective*; Oxford University Press: Oxford, U.K., 2000.
18. Blows, M.W.; Chenoweth, S.F.; Hine, E. Orientation of the genetic variance-covariance matrix and the fitness surface for multiple male sexually selected traits. *The American Naturalist* **2004**, *163*, 329-340.
19. Chenoweth, S.F.; Rundle, H.D.; Blows, M.W. The contribution of selection and genetic constraints to phenotypic divergence. *The American Naturalist* **2010**, *175*, 186-196.
20. Colautti, R.I.; Barrett, S.C.H. Population divergence along lines of genetic variance and covariance in the invasive plant *Lythrum salicaria* in eastern North America. *Evolution* **2011**, *65*, 2514-2529.
21. Bégin, M.; Roff, D.A. From micro- to macroevolution through quantitative genetic variation: positive evidence from field crickets. *Evolution* **2004**, *58*, 2287-2304.
22. Revell, L.J. Testing the genetic constraint hypothesis in a phylogenetic context: a simulation study. *Evolution* **2007**, *61*, 2720-2727.

23. Revell, L.J.; Harmon, L.J.; Langerhans, RB; Kolbe, J.J. A phylogenetic approach to determining the importance of constraint on phenotypic evolution in the neotropical lizard *Anolis cristatellus*. *Evolutionary Ecology Research* **2007**, *9*, 261-282.
24. Lynch, M. Methods for the analysis of comparative data in evolutionary biology. *Evolution* **1991**, *45*, 1065-1080.
25. Hadfield, J.D.; Nakagawa, S. General quantitative genetic methods for comparative biology: phylogenies, taxonomies and multi-trait models for continuous and categorical characters. *Journal of Evolutionary Biology* **2010**, *23*, 494-508.
26. Delahaie, B.; Charmantier, A.; Chantepie, S.; Garant, D.; Porlier, M.; Teplitsky, C. Conserved **G**-matrices of morphological and life-history traits among continental and island blue tit populations. *Heredity* **2017**, *119*, 76-87.
27. Meyer, K.; Houle, D. Sampling based approximation of confidence intervals for functions of genetic covariance matrices. *Proceedings of the Association for the Advancement of Animal Breeding and Genetics* **2013**, *20*, 523-526.
28. Houle D.; Meyer, K. Estimating sampling error of evolutionary statistics based on genetic covariance matrices using maximum likelihood. *Journal of Evolutionary Biology* **2015**, *28*, 1542-1549.
29. Morrissey, M.B.; Parker, D.J.; Korsten, P.; Pemberton, J.M.; Kruuk, L.E.B.; Wilson, A.J. The prediction of adaptive evolution: empirical application of the secondary theorem of selection and comparison to the breeder's equation. *Evolution* **2012**, *66*, 2399-2410.
30. Walling, C.A.; Morrissey, M.B.; Foerster, K.; Clutton-Brock, T.H.; Pemberton, J.M.; Kruuk, L.E.B. A multivariate analysis of genetic constraints to life history evolution in a wild population of red deer. *Genetics* **2014**, *198*, 1735-1749.
31. Sztepanacz, J.L.; Blows M.W. Accounting for sampling error in genetic eigenvalues using random matrix theory. *Genetics* **2017**, *206*, 1271-1284.
32. Thompson, R.; Cullis, B.; Smith, A.; Gilmour, A. A sparse implementation of the average information algorithm for factor analytic and reduced rank variance models. *Australian and New Zealand Journal of Statistics* **2003**, *45*, 445-459.

33. Kirkpatrick, M.; Meyer, K. Direct estimation of genetic principal components: simplified analysis of complex phenotypes. *Genetics* **2004**, *168*, 2295-2306.
34. Teplitsky, C.; Robinson, M.R.; Merilä, J. Evolutionary potential and constraints in wild populations. In: *Quantitative Genetics in the Wild*; Charmantier, A., Garant, D., Kruuk, L., Eds.; Oxford University Press: Oxford, U.K., 2014, pp. 190-208.
35. Robinson, M.R.; Beckerman, A.P. Quantifying multivariate plasticity: genetic variation in resource acquisition drives plasticity in resource allocation to components of life history. *Ecology Letters* **2013**, *16*, 281-290.
36. Ovaskainen, O.; Cano, J.M.; Merilä, J. A Bayesian framework for comparative quantitative genetics. *Proceedings of the Royal Society B-Biological Sciences* **2008**, *275*, 669-678.
37. Bishara, A.J.; Hittner, J.B. Testing the significance of a correlation with nonnormal data: Comparison of Pearson, Spearman, transformation, and resampling approaches. *Psychological Methods* **2012**, *17*, 399-417.
